# Gene Silencing of *BnTT10* Family Genes Causes Retarded Pigmentation and Lignin Reduction in the Seed Coat of *Brassica napus*

**DOI:** 10.1371/journal.pone.0061247

**Published:** 2013-04-22

**Authors:** Kai Zhang, Kun Lu, Cunmin Qu, Ying Liang, Rui Wang, Yourong Chai, Jiana Li

**Affiliations:** 1 Chongqing Rapeseed Engineering and Technology Research Center, Southwest University, Beibei, Chongqing, People's Republic of China; 2 Engineering Research Center of South Upland Agriculture, Ministry of Education, Southwest University, Beibei, Chongqing, People's Republic of China; 3 College of Agronomy and Biotechnology, Southwest University, Beibei, Chongqing, People's Republic of China; New Mexico State University, United States of America

## Abstract

Yellow-seed (*i.e.*, yellow seed coat) is one of the most important agronomic traits of *Brassica* plants, which is correlated with seed oil and meal qualities. Previous studies on the Brassicaceae, including *Arabidopsis* and *Brassica* species, proposed that the seed-color trait is correlative to flavonoid and lignin biosynthesis, at the molecular level. In *Arabidopsis thaliana*, the oxidative polymerization of flavonoid and biosynthesis of lignin has been demonstrated to be catalyzed by laccase 15, a functional enzyme encoded by the *AtTT10* gene. In this study, eight *Brassica TT10* genes (three from *B. napus*, three from *B. rapa* and two from *B. oleracea*) were isolated and their roles in flavonoid oxidation/polymerization and lignin biosynthesis were investigated. Based on our phylogenetic analysis, these genes could be divided into two groups with obvious structural and functional differentiation. Expression studies showed that *Brassica TT10* genes are active in developing seeds, but with differential expression patterns in yellow- and black-seeded near-isogenic lines. For functional analyses, three black-seeded *B. napus* cultivars were chosen for transgenic studies. Transgenic *B. napus* plants expressing antisense *TT10* constructs exhibited retarded pigmentation in the seed coat. Chemical composition analysis revealed increased levels of soluble proanthocyanidins, and decreased extractable lignin in the seed coats of these transgenic plants compared with that of the controls. These findings indicate a role for the *Brassica TT10* genes in proanthocyanidin polymerization and lignin biosynthesis, as well as seed coat pigmentation in *B. napus*.

## Introduction

*Brassica* species belong to the same taxonomic family Brassicaceae as *Arabidopsis*, representing the closest relatives to *Arabidopsis thaliana*. The genus *Brassica* contains many oilseed, vegetable and ornamental crops that are important sources of cooking oil, vegetables, and protein-rich meal for livestock feed. Among these species, *Brassica napus* is one of the most important oilseed crops cultivated, worldwide. The yellow-seeded *B. napus* varieties possess several advantages over black-seeded varieties, such as thinner seed coat, lower husk proportion and fiber content, and higher oil and protein content [1]–[3]. Extensive studies have established that the *B. napus* yellow-seed trait is highly correlated with high oil and meal qualities [1]–[3]. Unfortunately, currently, seed stock for the natural *B. napus* yellow-seeded genotype is unavailable. The yellow-seed trait was strongly influenced by environmental conditions [4]–[6]. Consequently, development of yellow-seeded *B. napus* cultivars and selection of stable yellow-seed traits has been a long-term breeding objective [7], [8]. However, a lack of information concerning the molecular basis controlling yellow-seed trait inheritance has seriously hampered progress in the breeding of yellow-seeded genotypes.

*B. napus* (2n = 38, AACC) is an amphidiploid species, originated from interspecific crosses of the diploid species *Brassica rapa* (2n = 20, AA) and *Brassica oleracea* (2n = 18, CC) [9]. The close phylogenetic relationship within these three *Brassica* species provides an ideal model for analyzing genetic evolution and practical implications for *Brassica* crop improvement by comparative studies. The high level of synteny and remarkably conserved genome structure between *Arabidopsis* and *Brassica* genomes [10] also enables comparative gene cloning and functional analysis in *Brassica* using *Arabidopsis* sequences as a reference.

A stable major quantitative trait locus (QTL) affecting seed coat color of *B. napus* in different generations and environments was earlier identified [7]. Based on microsynteny of this major QTL with *Arabidopsis* genome sequences, the functional gene *TT10* was considered as a potential candidate for this QTL. In *Arabidopsis*, the *AtTT10* (At5g48100) gene is one of 22 *TT*-type loci (*TT1-19*, *BAN*, *TTG1* and *TTG2*) identified by the *transparent testa* (*tt*) mutants that are affected in seed coat pigmentation, a process involved in flavonoid biosynthesis [11]–[14]. At harvest, seeds of the *tt10* mutant display pale-brown seed coat coloration with a dark-brown chalaza zone. After 6 to 12 months of storage, the seed coat turns brown and eventually it resembles the wild-type [15].

*AtTT10* encodes the laccase 15 (*AtLAC15*), an enzyme involved in polymerization of proanthocyanidin (PA) into larger polymers and probable further oxidation of PA, which confers brown color to the seed coat [15]. Additionally, *AtTT10* was also involved in lignin synthesis and polymerization of monolignols in *Arabidopsis* seeds [16]. PA was dramatically reduced in the seed coat of yellow-seeded rapeseed and seems to be the source of black-seed color in *B. napus* cv. Tower [17]. In *B. napus*, lignin content is an important trait of seed quality [8] and it is reported that yellow-seeded accessions, among the Brassicaceae, have significantly lower lignin content than those of brown- or dark-seeded accessions [17].

The correlation between PA polymerization and lignin synthesis with *B. napus* seed coat color, and the function of the *AtTT10* gene in seed coat pigmentation, strongly suggests that systematic cloning and functional identification of the *Brassica TT10* would provide a means to uncover the molecular mechanism of yellow-seed trait inheritance in *Brassica* species. In this study, 8 *Brassica TT10* genes were cloned from *B. napus* and its two parental species, *B. rapa* and *B. oleracea*. Expression studies and functional analyses performed on transgenic *TT10* gene-silenced plant lines established that *BnTT10* is involved in PA metabolism, lignin synthesis and seed coats pigmentation. These findings are discussed in terms of clues to the nature of the molecular mechanisms underlying the establishment of yellow seed traits in *Brassica* species.

## Materials and Methods

### Plant Materials and Nucleic Acid Extraction

Typical black-seeded *B. napus* line 5B, *B. rapa* line 06K130 and *B. oleracea* line 06K158 were used for gene cloning. *B. napus* NILs 09L588 (black-seed) and 09L587 (yellow-seed), *B. rapa* NILs 09L597 (black-seed) and 09L600 (yellow-seed), and *B. oleracea* NILs 09Bo-1 (black-seed) and 09Bo-4 (yellow-seed) were used for expression pattern detection of *Brassica TT10* genes by quantitative RT-PCR (qRT-PCR). Root (Ro), hypocotyl (Hy), cotyledon (Co), stem (St), leaf (Le), silique pericarp (SP) from each black-seeded line and bud (Bu), flower (Fl), seeds of 10 to 15, 25 to 30, 40 to 45 and 50 to 55 DAF of each black- and yellow-seeded lines were sampled for total RNA extraction using the CTAB method, with slight modifications [18]. Three independent plants from each line were sampled for RNA extraction. RNA aliquots were treated with RNase-free DNase I (TaKaRa, Dalian) to remove genomic DNA. Fresh leaves of each line were sampled to extract total genomic DNA, according to the CTAB method [19]. *B. napus* cv. Westar, Zhongyou821 and Zhongshuang10 were used for transgenic assays.

### Cloning of *TT10* Genes and Bioinformatics Analysis

The cDNAs of *Brassica TT10* genes were cloned by RACE with GeneRacer kit (Invitrogen, USA), according to manufacturer's instructions. For each of 5B, 06K130, and 06K158 lines, a 5-µg equally proportioned (w/w) mixture of total RNA extracted from Bu, Fl, and seeds sampled 10, 20, and 30 DAF was used for first-strand cDNA synthesis. Gene-specific primers were designed based on the conservative region of *AtTT10* and are listed in Table S1 [15], [20]. Primer combinations of RTT10-51, RTT10-52 and 5′-end cloning primers, and FTT10-31, FTT10-32 and 3′-end cloning primers were used for amplification of 5′- and 3′-ends of *TT10* genes. PCR products were cloned into the pMD18-T vectors (TaKaRa, Dalian) and sequenced. The cDNAs of *Brassica TT10* genes were amplified with combinations of 5′ primers (FBNTT10, FBRTT10 and FBOTT10) and 3′ primers (RBNTT10, RBRTT10, RBOTT10 and RBRTT10). Primers successful in cDNA amplifications were used to amplify corresponding genomic DNA sequences.

Open reading frame (ORF), parameter calculation and sequence alignment were performed with Vector NTI Advance 10.3 (Invitrogen). Sequence alignments and protein structure predictions were performed on Expasy (http://www.expasy.org). Multiple sequence alignments results from Clustal ×1.83 were subjected for phylogenetic tree construction by the Neighbor-Joining method with MEGA 3.1 [21], [22]. The reliability of the tree was measured by bootstrap analysis with 1,000 replicates.

### Southern Hybridization

For each *Brassica* species, 50-µg genomic DNA was fully digested at 37°C with *Dra*I, *Eco*RI and *Eco*RV (Fermentas, Lithuania), respectively, separated in 0.8% agarose gels, and transferred onto positively charged nylon membrane (Roche, Germany). A 951-bp fragment, mainly composed of the fifth exon of *TT10* and no cutting sites of restriction enzymes for genomic DNA digestion, was used as probe, which was then amplified with primers FTT10A and RTT10A using *BnTT10-2* cDNA as template. Probe labeling was carried out using a PCR DIG Probe Synthesis Kit (Roche) and hybridization was then performed with the DIG Easy Hyb Kit (Roche), and immunological detection was implemented with DIG Wash and Block Buffer Set and DIG Nucleic Acid Detection Kit (Roche), according to the manufacturer's instructions.

### Expression Pattern Assay

Expression patterns of *TT10* genes in various organs of three *Brassica* species were detected by qRT-PCR. One µg of each total RNA sample was reverse-transcribed in a 10-µl volume, using RNA PCR Kit (AMV) Ver.3.0 (TaKaRa, Dalian) and 0.5 µl of the RT product was used as template for a 25-µl standard *Taq*-PCR. A 193-bp conserved region of *18S rRNA* was amplified as an internal control [23]. Primers for qRT-PCR were: FBnT10Q and RBnT10Q for detection of overall *TT10* gene expression; FBnT10-1Q and RBnT10-1Q for detection of *BnTT10-1* and *BrTT10-1A*; FBnT10-2Q and RBnT10-2Q for detection of *BnTT10-2* and *BoTT10-1*; FBnT10-3Q and RBnT10-3Q for detection of *BnTT10-3* and *BRTT10-2*; FBrT10-1BQ and RBrT10-1BQ for detection of *BrTT10-1B*; and FBoT10-1pseQ and RBoT10-1pseQ for detection of *BoTT10-1pse*. Reactions were performed in triplicate from 3 independent samples, with a negative water control in each run. The specificity of qRT-PCR products was confirmed by agarose gel electrophoresis followed by sequencing.

### Transgenic Plant Development

The 951-bp conserved fragment used for Southern hybridization was cloned in antisense orientation into the binary vector, pCAMBIA2301G. This antisense *TT10* expression construct was transferred into *B. napus* cv. Westar, Zhongyou821 and Zhongshuang10 using *Agrobacterium tumefaciens* strain LBA4404, as previously described [24].

GUS staining and PCR identification of leaf pieces was used for selection of T_0_ generation transgenic plants [25]. To assess the inhibition of *BnTT10* gene expression in seeds of transgenic plants, qRT-PCR was applied to measure the overall *TT10* and individual genes *BnTT10-1*, *BnTT10-2* and *BnTT10-3* expression in seeds at 35 DAF with the above-mentioned gene-specific primers. Negative plants were kept as controls.

### Seed Pigmentation Observation

To observe seed coat color during seed development, pods were regularly sampled at 40, 45, 50, 55 and 60 DAF, and the stripped pods were visualized under a low-power stereoscope. For observation of seed coat color during seed after-ripening, pods sampled at 40, 50, and 55 DAF were also stripped and surveyed after 5 and 15 days of *in vitro* storage at 4°C.

### Quantification of Proanthocyanidin (PA) and Extractable Lignin Content

Soluble and insoluble PA content was determined using the butanol-HCl method, as previously described [16], [26]. Assessment of extractable lignin content was carried out according to the acetyl bromide method, as previously described [27], [28], with slight modifications. The assessment was conducted using three independent batches of seed samples. Each sample was assayed in triplicate to obtain a mean value. Proanthocyanidin and extractable lignin content are shown in absorbance value (OD_550_ and OD_280_, respectively) per sample weight unit.

### High Performance Liquid Chromatography (HPLC)-MS-UV Analysis

Liquid chromatography-mass spectrometry (LC-MS) solvents were obtained from Fisher Science (Rockford, IL, USA). Flavonoid standards were obtained from Indofine (Somerville, NJ, USA), Sigma-Aldrich (St. Louis, MO) and Chromax (Irvine, CA). Freshly harvested seed coats (100 mg fresh weight) were homogenized in 80% methanol (1 ml), and the suspension was placed in an ultrasonic bath for 1 h. The extract was centrifuged (1.0×10^4^ g, 20 min) and the supernatant was then filtered.

Analyses of plant extracts were performed with an Agilent 1100 HPLC system (Hewlett-Packard, Palo Alto, CA) combined with an ion trap mass spectrometer (model Bruker Esquire 3000, Bruker Daltonics, Bremen Germany). Instrument analyses were carried out using a Grace Column (2.0×250 mm, grain diameter 4.6 µm). UV-visible spectra were obtained by scanning from 200 to 600 nm. The mobile phase consisted of (A) water containing 0.1% (v/v) formic acid and (B) acetonitrile, using the following binary gradient: 0 to 5 min, isocratic 95% A and 5% B; 5 to 10 min, isocratic 10% B; 10 to 17 min, isocratic 17% B; 17 to 25 min, isocratic 25% B; 25 to 30 min, isocratic 30% B, 30 to 55 min, isocratic 55% B, 55 to 65 min, isocratic 70% B. 65 to 70 min, isocratic 5% B, 70 to 75 min, isocratic 95% A and 5% B. Flow rate was 0.8 ml min^−1^. Negative-ion ESI mass spectra were employed, using an ion source voltage of 3.5 kV, a counter current nitrogen flow set at a pressure of 12 psi, and a capillary temperature of 350°C. Mass spectra were recorded over the range 50–2,200 m z^−1^. The Bruker ion-trap mass spectrometer (ITMS) was operated using an ion current control (ICC) of approximately 1×10^4^ with a maximum acquisition time of 100 ms. Tandem mass spectra were obtained in manual mode for targeted masses using an isolation width 2.0, fragmentation amplitude of 2.2 and threshold set at 6×10^3^.

Accession Numbers: HM805058 (*BnTT10-1* gene), HM805059 (*BnTT10-1* mRNA), HM805060 (*BnTT10-2* gene), HM805061 (*BnTT10-2* mRNA), HM805062 (*BnTT10-3* gene), HM805063 (*BnTT10-3* mRNA), HM805064 (*BrTT10-1A* gene), HM805065 (*BrTT10-1A* mRNA), HM805066 (*BrTT10-1B* gene), HM805067 (*BrTT10-1B* mRNA), HM805068 (*BrTT10-2* gene), HM805069 (*BrTT10-2* mRNA), HM805070 (*BoTT10-1* gene), HM805071 (*BoTT10-1* mRNA), HM805072 (*BoTT10-1pse* mRNA).

## Results

### Cloning and Sequence Analysis of *TT10* Genes from *B. napus* and Its Two Parental Species

Three full-length cDNAs, designated as *BnTT10-1*, *BnTT10-2* and *BnTT10-3*, were cloned from *B. napus* (Fig. S1), two full-length cDNAs *BoTT10-1*, *BoTT10-1pse* were isolated from *B. oleracea*, and three full-length cDNAs *BrTT10-1A*, *BrTT10-2*, *BrTT10-1B* were obtained from *B. rapa* (Table S2). Corresponding genomic DNA of these cDNAs were obtained by PCR amplification with genomic DNA as template. Unfortunately, genomic DNA sequences for *BnTT10-2* and *BoTT10-1pse* were not obtained.

Southern blot hybridization against genomic DNA of *B. napus*, *B. oleracea* and *B. rapa* showed 4, 1 and 1–2 bands in all lanes, respectively (Fig. S2), indicating the presence of minimum number of *TT10* genes. Taken together, our results of gene cloning and Southern blot hybridization suggest that the eight cloned *Brassica TT10* genes comprise the majority of *TT10* genes in *B. napus* and its two parental species.

Based on transcript sequence similarity and the theoretical protein translations, the *Brassica TT10* genes could be divided into two groups (Table S3 and S4). Group I included *BnTT10-3* and *BrTT10-2* that share 99.7% similarity and encode identical proteins of 560 amino acids (aa), Group II contains the remaining six Brassicaceae *TT10* genes with similarity of 94.6% to 99.8%, whereas their similarity to Group I members was only 85.2% to 86.3%. Among Group II, *BnTT10-1, BrTT10-1A* and *BrTT10-1B* showed 99.3% to 99.8% identity at the mRNA level, and the deduced proteins of the three members were all 559 aa in length and shared 99.3% to 99.8% similarity. *BnTT10-2* and *BoTT10-1* have identical cDNA sequences and encode identical proteins of 563 aa; *BoTT10-1pse* does not encode a fully functional protein, due to deletion mutations leading to a frame-shift and premature termination of translation. Pairwise alignment indicates that either cDNA or protein sequences of *Brassica TT10* genes are approximately 80% identical to that of *AtTT10*.

SignalP 3.0 (http://www.cbs.dtu.dk/services/SignalP/) predicted that BnTT10 proteins contain a signal peptide most likely cleaved at A_21_–H_22_. PSORT (http://psort.ims.u-tokyo.ac.jp/) also predicted that Group I proteins would be secreted to the cell wall. In contrast, Group II proteins appear to have an uncleavable N-terminal signal sequence and may well be endoplasmic reticulum-located proteins. *Brassica* TT10 proteins were as conserved as AtTT10 and other laccases. In addition, as previous reported, they contain four His-rich copper binding domains, L_1_ to L_4_ [29], and two signatures of the multicopper oxidase family, the 21-aa multicopper oxidase signature 1 and 12-aa signature 2 (Fig. S3), corresponding to the putative catalytic sites conserved in laccases [29]. Phylogenetic analysis indicated that these *Brassica* TT10 proteins (excluding BoTT10-1pse) formed a small branch clustered with AtTT10 and diverged from other analyzed laccases (Fig. S4).

### Spatial and Temporal Expression Patterns of *Brassica TT10* Genes

**Figure 1:**
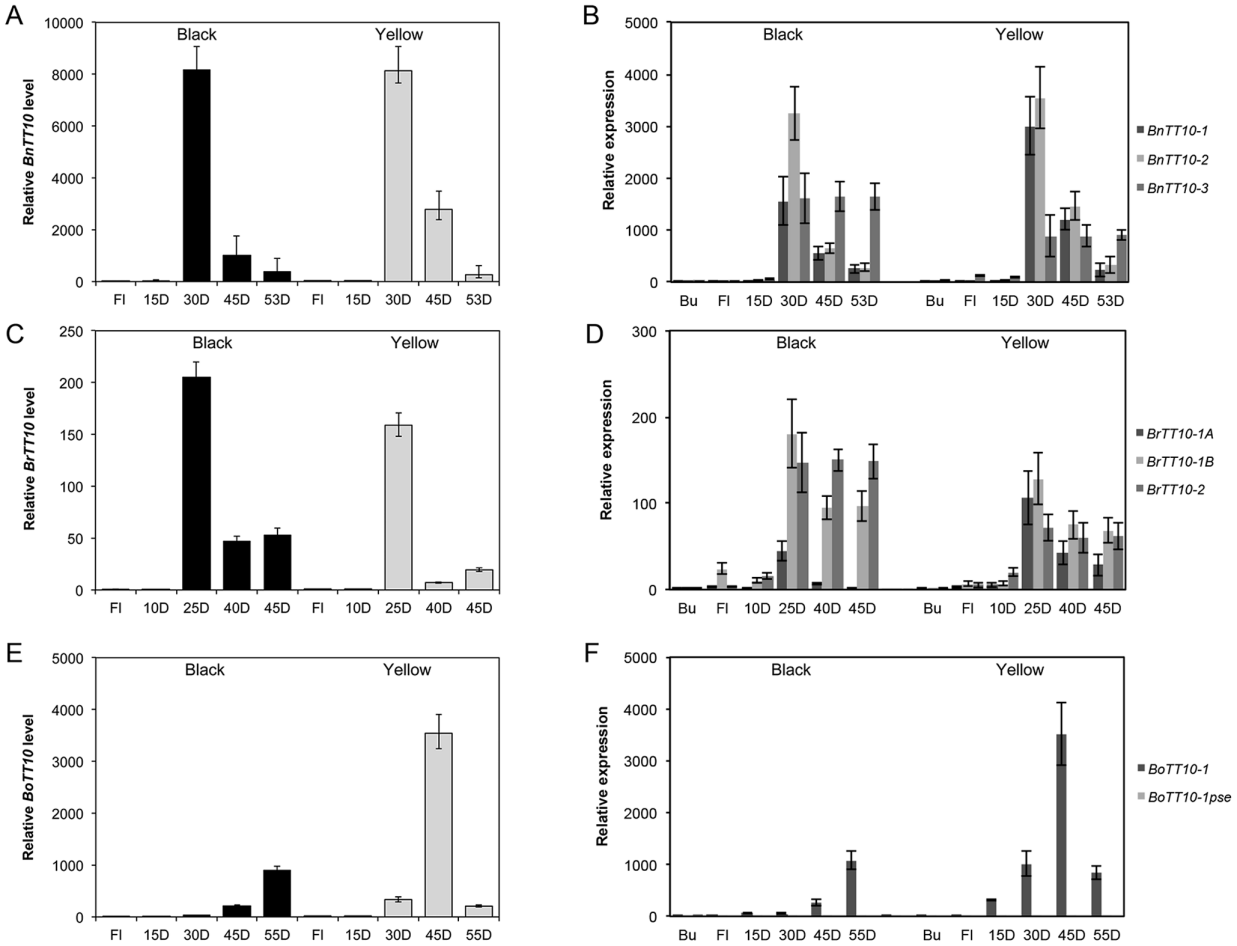
Expression patterns of *BnTT10*, *BrTT10* and *BoTT10* genes in black- and yellow-seeded lines. QRT-PCR detection of *BnTT10* (A), *BrTT10* (C) and *BoTT10* (E) family mRNA, and transcript levels of *BnTT10* (B), *BrTT10* (D) and *BoTT10* (F) family members in reproductive organs of *B*. *napus*, *B. rapa* and *B. oleracea* black- and yellow-seeded near-isogenic lines. Error bars represent standard deviations (*n* = 3 for A, C and E).

*AtTT10* was mainly expressed in developing siliques and seeds, specifically in the seed coat [15], [16], [20], [30]. To verify the organ-specific function of *Brassica TT10* genes, qRT-PCR was used to determine their mRNA levels in different organs of the black-seeded lines from the three *Brassica* species. As shown in Fig. 1 and S5, *Brassica TT10* genes were predominantly expressed in developing seeds, low level transcripts of *BnTT10-3*, *BrTT10-2* and *BrTT10-1B* were observed in flowers, whereas expression of *TT10* genes was hardly detected in silique pericarp, hypocotyl, stem or root tissues. Transcripts for *BoTT10-1pse*, the *B. oleracea TT10* gene with deletion mutations, was undetectable in all organs examined. We also found that *BnTT10* genes were expressed at very low levels in hypocotyls and roots; here, expression was almost 5×10^−4^ below that in seeds.

Temporal expression of *Brassica TT10* genes was analyzed with developing seeds from the three *Brassica* species. The Group I members, *BnTT10-3* and *BrTT10-2*, showed similar expression patterns through middle (25 to 30 days after flowering [DAF]), late (40 to 45 DAF) and pigmented (45 to 53 DAF) stages (Fig. 1 and S5). The Group II members, *BnTT10-1*, *BnTT10-2* and *BrTT10-1B*, were found to be expressed only weakly at the early stage, at highest levels during the middle stage, and strongly at late and pigmented stages. *BrTT10-1A* was expressed strongly at middle stage, moderately at late stage, but transcripts were undetectable at the early and weakly in pigmented stages. Expression of *BoTT10-1* showed a seed-development-stage-dependent increase with weakest expression during the early stage and highest expression at the pigmented stage (Fig. 1 and S5).

### Correlation Between *TT10* Expression and Yellow-seed Trait of *B. napus* and Its Two Parental Species

To examine the correlation between yellow-seed trait and *TT10* gene expression in *Brassica* species, expression of *Brassica TT10* genes was detected in developing seeds and flowers from black- and yellow-seeded lines of the three *Brassica* species. In developing seeds of *B. rapa* and *B. oleracea*, yellow-seeded lines showed down- and up-regulation of overall expression of *TT10* genes compared with the black-seeded lines, respectively (Fig. 1C and 1E). The yellow-seeded lines of *B. napus* showed slight up-regulation of overall expression of *TT10* genes in seeds (45 DAF) compared with the black-seeded lines (Fig. 1A). Moreover, individual members also showed different expression patterns (Fig. 1B, 1D and 1F). Group I genes (*BnTT10-3*, *BrTT10-2*) and a Group II member (*BrTT10-1B*) showed higher expression levels in developing seeds of black-seeded lines compared to yellow-seeded lines, whereas transcription of other Group II members (*BnTT10-1, BnTT10-2*, *BrTT10-1A* and *BoTT10-1*) exhibited the opposite trend. Additionally, in developing seeds of *B. oleracea*, *BoTT10-1* showed the highest expression level in pigmented (55 DAF) stage of black-seeded lines, but in late (45 DAF) stage of yellow-seeded lines (Fig. 1F).

### Antisense Suppression of *TT10* Genes Retards Pigmentation in the Seed Coat of Black-Seeded *B. napus*

To investigate the function of *Brassica TT10* genes in seed coat pigmentation, a 951-bp DNA fragment conserved in the *Brassica TT10* genes was expressed in antisense orientation to suppress *TT10* genes in black-seeded *B. napus*. A total of 14, 8 and 9 T_0_ plants of Westar, Zhongyou821 and Zhongshuang10 were identified as positive transgenic plants by GUS staining and qRT-PCR analysis, in which the overall expression of *TT10* genes was repressed up to 83% (Fig. S6), and suppression level of individual members were similar (Fig. S6). For each cultivar, three T_0_ plants with suppressed *TT10* expression were selected to generate progenies for seed coat pigmentation analysis.

**Figure 2:**
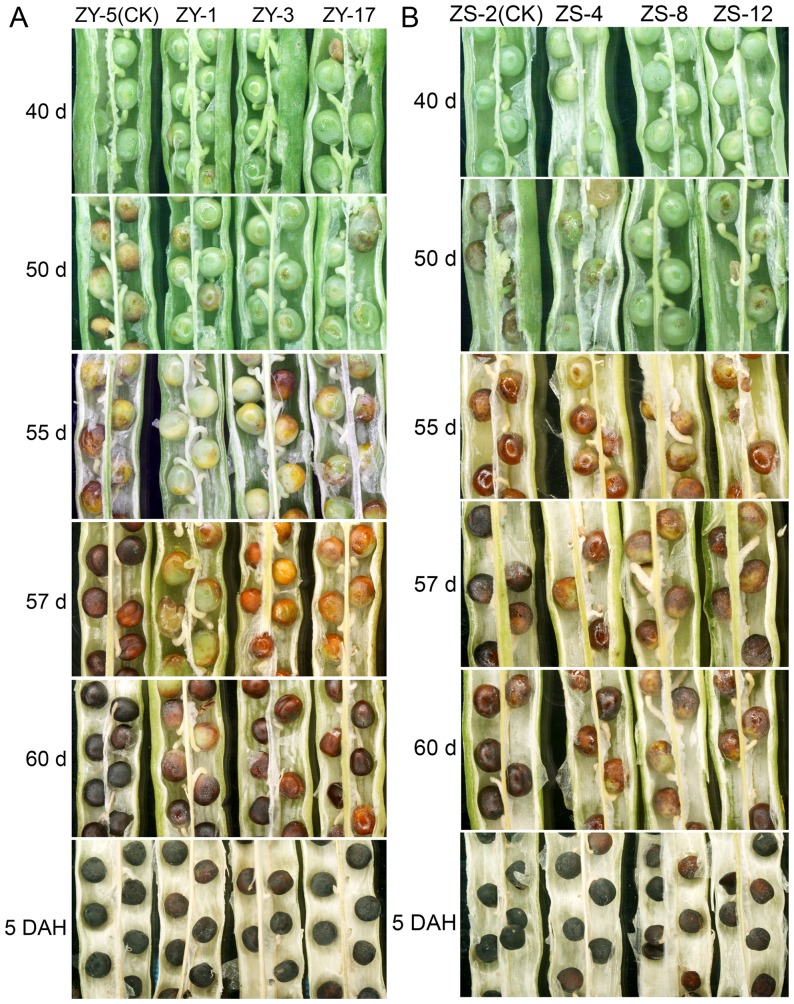
Seed pigmentation observation. Seedpods were sampled at 42, 50, 55, 60 and 45 DAF and the opened pods were observed under a low-power stereoscope. 5 DAH: five days after harvest. Seed coat pigmentation in the T_2_ transgenic and control *B. napus* cv. Zhongyou821 (A) and Zhongshuang10 (B).

Seeds of T_2_ generation from Zhongyou821 and Zhongshuang10 transgenic plants exhibit various degrees of retarded seed coat pigmentation at the maturing stage when compared with control plants (Fig. 2). To explore the association of autoxidation with seed pigmentation, the *in vitro* seed pigmentation process of transgenic plants was also investigated. When pods were picked at 40 DAF, seed pigmentation of control plants was initiated rapidly and the seed coat color turned to black in 5 days of storage (DS) (Fig. S7). In contrast, under these conditions, seeds of transformants showed retarded pigmentation after 5 DS (Fig. S7) and they displayed homogeneous red-brown color after 15 DS, with no trend to turn black after a month of storage under ambient temperature conditions.

### Suppression of *TT10* Genes Increased Soluble PAs and Resulted in Reduction of Lignin Content in Seed Coats

**Figure 3:**
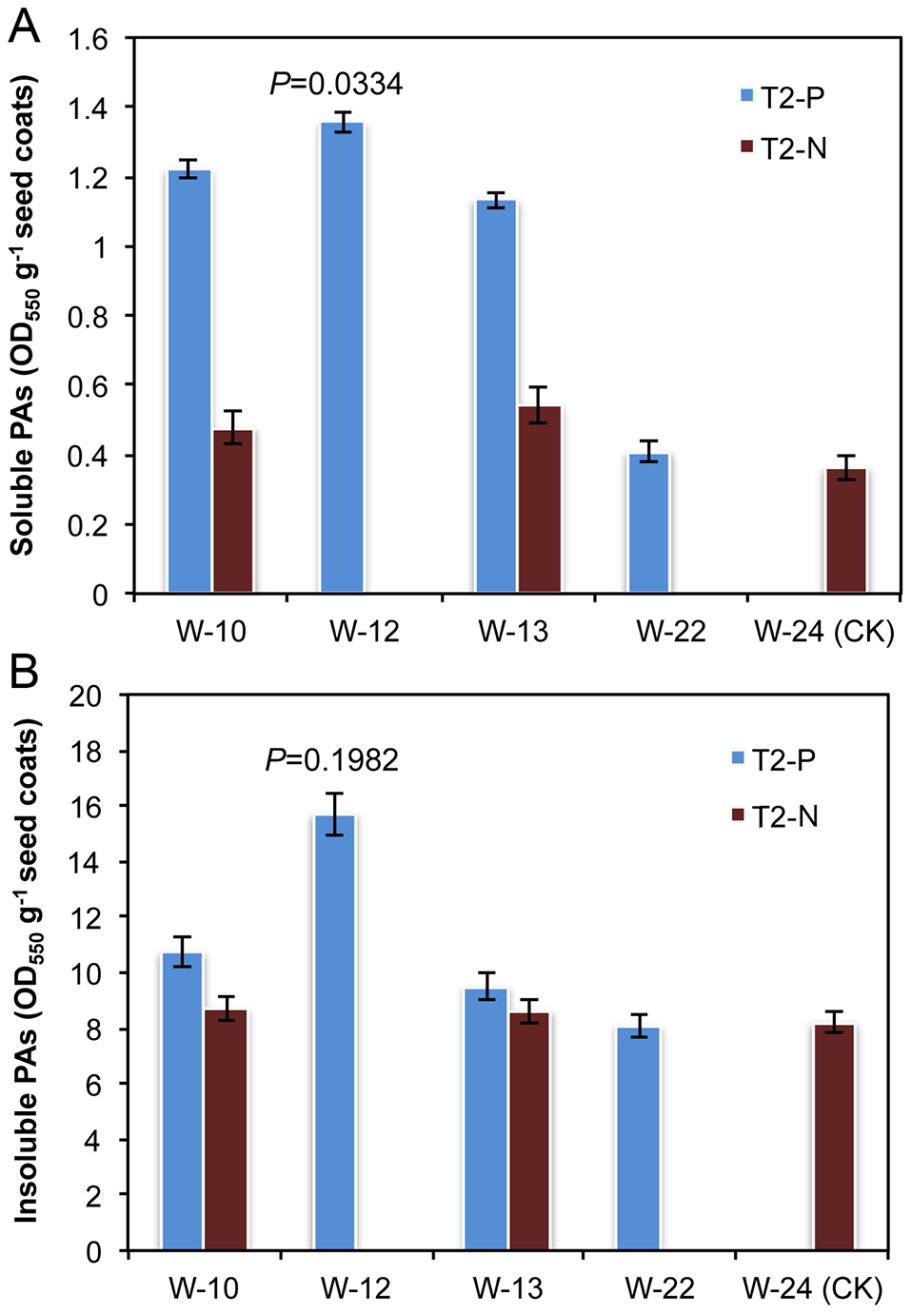
Soluble and insoluble PAs measured after acid-catalyzed hydrolysis in seed coats transgenic and control lines. The *P*-value is for a *t*-test for means of paired samples. W-10, W-12, W-13: transgenic lines with inhibited *BnTT10* expression; W-22: transgenic lines with no inhibition in *BnTT10* expression; W-24: control lines with normal *BnTT10* expression. T_2_-P: positive T_2_ progenies; T_2_-N: negative T_2_ progenies after separation. Soluble (A) and insoluble PA (B) content in seed coats of T_2_ transgenic and control *B. napus* cv. Westar plants. Each value represents the means of three independent experiments +/− SD.

To understand the roles of *Brassica TT10* genes in PA polymerization, we measure the PA content in seed coats of transgenic *B. napus*. The acetone-soluble PA content in seed coats of T_2_ transgenic Westar plants was more than 3 fold higher than that detected in the control, and this increase was highly correlated with the degree to which the *TT10* transcripts were lowered in these transgenic lines (Fig. 3A). Interestingly, the acetone-insoluble PA of seed coats, measured in the remaining residue after solvent extraction, also showed 1.2 to 1.9 fold increase in these transgenic Westar lines compared with the control (Fig. 3B). However, here, the difference of insoluble PA content was smaller than for soluble PA-content.

**Figure 4:**
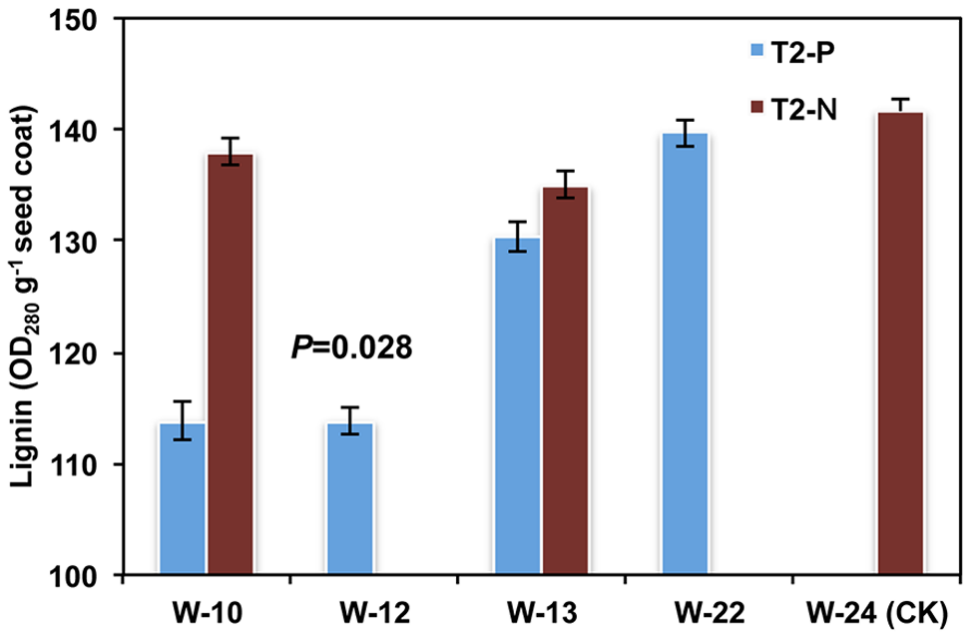
Lignin content in seed coats of transgenic and control lines. Lignin content was analyzed for seed coats of T_2_ antisense *BnTT10* transgenic and control lines using the acetyl bromide method. Data are means for three T_2_ progenies of each line, with triplicate measurements in each sample. The *P*-value is for a *t*-test for means of paired samples. W-10, W-12 and W-13: transgenic lines with inhibited *BnTT10* expression; W-22: transgenic lines with no inhibition in *BnTT10* expression; W-24: control lines with normal *BnTT10* expression. T_2_-P: positive T_2_ progenies; T_2_-N: negative T_2_ progenies after separation. Each value represents the means of three independent experiments +/− SD.

Seed coats of T_2_ transgenic lines contained less extractable lignin than the controls, being decreased by 5%, 12% and 16% in the three transgenic lines (Fig. 4). Transgenic line W-12 exhibited close to 83% reduction in overall *TT10* mRNA level and showed the highest decrease in lignin content compared with the other lines. Consistent with this notion, soluble and insoluble PA content accumulation (Fig. S8 and S9), and reduction in lignin content in seed coats was also verified in Zhongyou821 and Zhongshuang10 transgenic lines (Fig. S10 and S11).

Negative T_2_ progenies generated from positive T_1_ plants and the transgenic lines without repression of *TT10* gene expression (W-22) showed no significant difference with the control with respect to PA and lignin content of the seed coat. This strengthened our conclusion that the observed decrease in PA content and reduction in lignin content in seed coats from transgenic plants was due to a reduction in the transcript levels for the *TT10* genes.

### Suppression of *TT10* Genes Changed Flavonoid Composition in the Seed Coat

**Figure 5:**
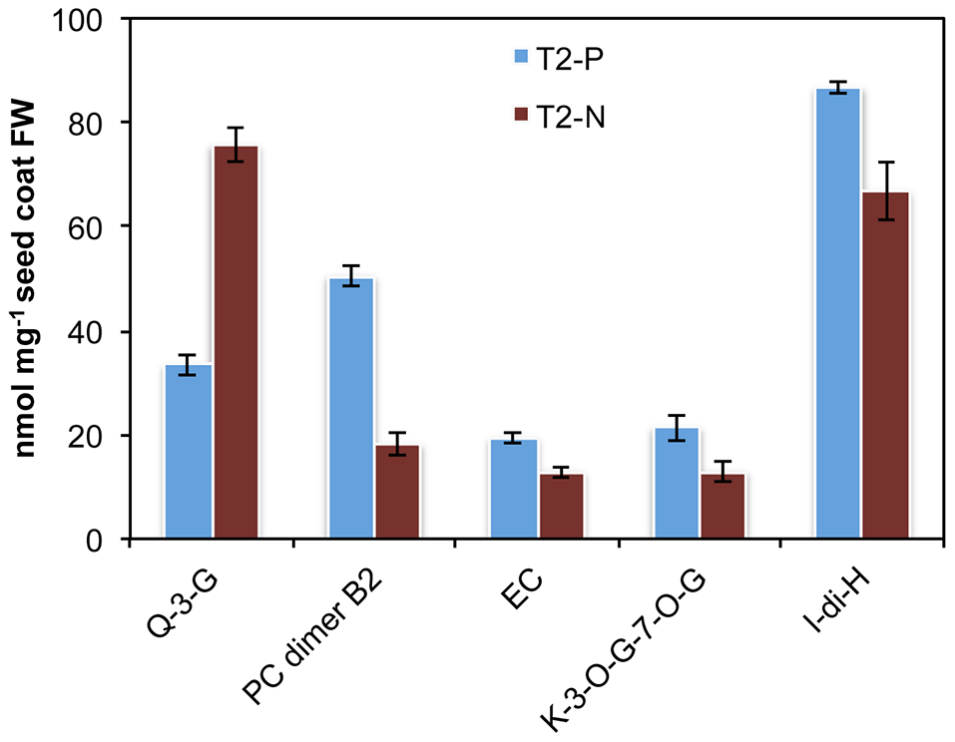
Detection of flavonoid composition in seed coats from transgenic and control *B. napus* plants. Analyses were performed by LC-UV-MS on seed coats of T_2_ antisense *BnTT10* transgenic and control lines of *B. napus* cv. Zhongyou821. Q-3-G, Quercetin-3-glucoside; PC dimer B2, [DP2]-B2, epicatechin-(4β-8)-epicatechin; EC, epicatechin; K-3-O-G-7-O-G, kaempferol-3-O-glucoside-7-O-glucoside; I-di-H, isorhamnetin-dihexoside. Each value represents the means of three independent experiments +/− SD.

Epicatechin and procyanidin polymers were detected by liquid chromatography- mass spectrometry (LC-MS) analysis of the soluble fractions (Fig. 5 and S12). Compared with seed coats from control plants, monomer PA epicatechin and procyanidin dimer B2 (epicatechin-[4β-8]-epicatechin) were 1.5 and 2.8 fold more abundant in transgenic lines with reduced *BnTT10* transcript levels. We also carried out LC-MS analysis of anthocyanins and/or other flavonoids. Seed coats of transgenic lines contained more kaempferol-3-O-glucoside-7-O-glucoside and isorhamnetin-dihexoside compared with the control. On the other hand, quercetin-3-glucoside was about 2.3 fold lower than in the control seed coats. The abundance of other flavonols was not significantly changed by the antisense suppression of *TT10* genes (Fig. 5).

## Discussion

### *Brassica TT10* Genes Show Differential Involvement in the Yellow Seed Trait

Our studies established that the *BrTT10* and *BoTT10* genes are donors of *BnTT10* genes. Highly homologous mRNA sequences, identical protein sequences and similar expression patterns indicated that *BrTT10-1A, BoTT10-1* and *BrTT10-2* are progenitors of *BnTT10-1, BnTT10-2* and *BnTT10-3*, respectively. Moreover, individual *BnTT10* genes probably inherited the expression patterns of its donor gene, for spatial and temporal characteristics, as well as association with the yellow seed trait. *BnTT10-2* and its donor gene, *BoTT10-1*, *BnTT10-1* and its donor gene, *BrTT10-1A*, showed higher expression in yellow-seeded lines, whereas *BrTT10-2* and its acceptor gene, *BnTT10-3*, showed down-regulated expression in yellow-seeded lines, indicating that individual gene members of a gene family may play individual roles in terms of the yellow seed trait. However, overall *TT10* expression in developing seeds of yellow- and black-seeded near-isogenic lines (NILs) of *B. napus* showed no significant difference. Therefore, the features of amphidiploid *B. napus* and the *TT10* multi-gene family should be considered in studies to elucidate the molecular basis of yellow seed traits.

### Involvement of *BnTT10* Genes in PA Accumulation in Seed Coats

PAs are oligomers and polymers of flavan-3-ols ((+)-catechin and (–)-epicatechin) units, also called condensed tannins, and known as end products of the flavonoid biosynthetic pathway [31], [32]. Based on studies in *Arabidopsis*, it has been proposed that *AtTT10* functions in PA polymerization [15], [16]. Our result also showed that suppression of *TT10* genes results in an increase in the level of soluble PAs (i.e. monomers or oligomers of PAs) in seed coat, indicating that *BnTT10* probably performs the same function as *AtTT10* at the step of oxidative polymerization of PAs. Our LC-MS analysis confirmed this result. Seed coats of transgenic plant lines accumulated more epicatechin and PA dimer B2 (epicatechin-[4β-8]-epicatechin) than that of controls, suggesting that *BnTT10* genes are involved in polymerization of these two types of soluble PAs, the monomer and B2-type dimer epicatechin.

In addition, the PA content in the seed coat residue remaining after solvent extraction was also higher in the transgenic lines compared with the control. These results are similar to the findings of Pourcel et al. [15] and Liang et al. [16], in that *tt10* mutant seeds showed increases in total PA content. According to the report of Marles and Gruber [17], PA was tightly bound in mature Brassicaceae seed coats and only a very small amount of PA could be extracted from the seed coat of these species and detected as released anthocyanidin subunits. Thus, the increase in PA content, in the seed coat residue, can be regarded as a decrease in larger polymers, which could not be acid hydrolyzed. Presumably, then, BnTT10 is able to further oxidize PAs into larger polymers in seed coat of *B. napus*.

Our studies indicated that functional divergence occurred between the two groups of *Brassica TT10* genes in PAs oxidative polymerization. Group II members, excluding *BoTT10-1* and *BoTT10-1pse*, achieved their highest level of expression in middle-stage (25 to 30 DAF) seeds, with a decreasing trend during the gradual seed maturing process. In addition, the predicted subcellular localization of Group II proteins is the endoplasmic reticulum (ER) and in ER-derived vesicles in which flavan-3-ols are synthesized as colorless polymers on *Arabidopsis* testa [15], [33], [34]. In this scenario, Group II genes probably act at the early step of PA polymerization, though the product may not be colorless [15]. In contrast, the Group I members, *BnTT10-3* and *BrTT10-2*, exhibited strong expression during the entire process of seed development. As with *AtTT10*, the Group I genes are predicted to be secreted into the apoplast, where epicatechin and PAs would interact with TT10 proteins to become oxidized and polymerized during the seed desiccation period [15], [35]. Hence, it is possible that the *TT10* Group I genes participate primarily in the further oxidation of PAs.

### *TT10* Genes Affect the Pigmentation Process of Seed Coats in *B. napus*

In brown or dark seed coats, the pigmentation process is thought to be initiated by the polymerization of flavan-3-ol monomers into colorless PAs polymers, which then undergo successive oxidation reactions leading to more complex oxidation levels of PAs during seed maturation. The final product is the insoluble complexes within the cell wall, with the resultant browning or darkening of the seed coat [4], [15]. *TT10* has been shown to catalyze the oxidative polymerization of epicatechin into yellow to brown PA derivatives that differ from the colorless PAs in *Arabidopsis* [15], our study also established that *BnTT10* genes act probably at the step of polymerization of monomers or oligomeric PA into polymers, thus, the functional defect caused by our transgenic mediated reduction in *BnTT10* transcript level may affect the accumulation of pigments in seed coats and, furthermore, caused the observed delay in the onset of discoloration.

Our results establish that the *BnTT10* play a key role in the initiation of seed coat browning or darkening in *B. napus*. This finding is also corroborated by *in vitro* observations of seed pigmentation. Compared with seed pigmentation of control plants, seeds of transgenic plants, harvested at 40 DAF (here, seed coat pigmentation has not yet been initiated), also showed pigmentation delay after 5 days storage at 4°C. The fact that seed coat color did not turn black, after a month of after-ripening, revealed that the role of *BnTT10* in pigment precursor accumulation within the seed coat could not be compensated for by another enzymatic reaction and autoxidation. Then, seeds from transgenic plants, harvested at 50 DAF and 55 DAF (late stage of pigmentation), became completely discolored during a 5-day-storage period, implying that there is enough pigment precursors had accumulated in the seed coat and are readily oxidized to form pigment that confers browning or darkening of the seed coat, and then, the seed then accomplished pigmentation by autoxidation (a condition resembling the after-ripening process of rapeseed). Given that PA polymers may have multiple stereochemistries [31], the forms of the pigments associated with dark seed coat coloration need to be studied to determine the role of both enzymatic reaction and auto-oxidation in the further oxidation of PA polymers.

### Involvement of *BnTT10* Genes in Lignin Synthesis in Seed Coats

One of the functions of plant laccases is in the polymerization of monolignol to lignin polymers, which are constituents of the plant cell wall [36], [37]. It has been reported that laccases are involved in lignin synthesis [36], [38], and Liang et al. [16] showed that lignin content in mature seeds of the *Arabidopsis tt10* mutant is decreased by some 30% and, further, developing seeds of the *tt10* mutant also have reduced activity in terms of monolignol polymerization. Consistent with a role for *BnTT10* genes in lignin sysnthesis, seed coats of transgenic *B. napus* plants had reduced levels of lignin. Although lignin content was reported to be significantly lower in yellow-seeded samples compared with the dark-seeded accessions, and low lignin is strongly associated with the unpigmented seed coat trait in Brassicaceae [17], the correlation of lignin synthesis with formation of seed coat color needs to be further investigated. Since lignin content is an important trait that needs to be considered when selecting for low-phenolic and low-fibre rapeseed [17], *BnTT10* genes may provide an avenue for further quality improvement.

### *TT10* May Not Be the Major Locus for the Yellow-Seed Trait

The seed coat of yellow-seeded rapeseed is uncolored and the associated degree of transparency allows the underlying yellow-colored embryo to be visible. Although the transgenic plants with suppressed *BnTT10* expression showed reduced seed pigmentation, the T_2_ progeny of these transgenic lines showed no difference in seed coat color with that of the control plants. Furthermore, the seed coat of the *Arabidopsis tt10* mutant has an unstable pale brown color, but is not completely colorless. Thus, neither a complete nor incomplete functional deficiency of the *BnTT10* genes would give rise to yellow seed color in *B. napus*. Taken together, these findings suggest that the *BnTT10* genes may not function as the major locus that is crucial for development of the yellow-seed trait.

Based on previous studies from our group, the yellow-seed trait may represent a QTL that is controlled by a combination of a major gene and several minor genes. Considering that lignin and PA synthesis are two diverged branches of the phenylpropanoid metabolic pathway, in conjunction with our finding that both extractable and unextractable PA, as well as lignin content, are dramatically reduced in seed coats of yellow-seeded rapeseed [17], [39], the possibility exists that the major gene responsible for the yellow-seed trait is an upstream gene acting on a common step, or affecting the accumulation of a common precursor of PA and lignin synthesis. Alternatively, as genes acting at the terminal steps of the two branches, *BnTT10* may be one of the downstream genes regulated by this major gene of the yellow-seed trait. Investigations on biosynthesis and deposition of pigments and lignin, within the seed coat, may provide further insight into the molecular basis of this important trait.

## Supporting Information

Figure S1 **Alignment of the mRNA sequences of** ***BnTT10*** **genes.** Conservative similar residues were displayed in dark background. The 951-bp conserved fragment used for Southern hybridization and antisense suppression was highlighted by red line.
(PDF)

Figure S2 **Southern blot hybridization detection of** ***TT10*** **genes in** ***B. napus*** **,** ***B. oleracea*** **and** ***B. rapa*** **.**
(TIF)

Figure S3 **Alignment of the amino acid sequences of BnTT10 proteins.** BrTT10 and BoTT10-1 proteins share high identity with BnTT10 proteins and were not shown in the alignment. Non-similar, weakly similar, block of similar, conservative/strongly similar residues are displayed in dark, dark gray, gray and light gray background, respectively. The four His-rich copper binding domains, L_1_-L_4_, are highlighted by line segments, and the amino acids involved in binding copper are boxed. Two predicted multicopper oxidase signature 1 and 2 are identified by single and double dotted lines, respectively.
(TIF)

Figure S4 **Phylogenetic relationship of inferred** ***Brassica*** **TT10 proteins and other plant laccases.** *Acer pseudoplatanus* ApLAC1 (AAB09228); *Aspergillus flavus* AfLAC (XP_002378028); *Arabidopsis thaliana* AtTT10 (NP_199621), AtLAC4 (NP_565881), AtLAC12 (NP_196158), AtLAC13 (NP_196330), AtLAC14 (NP_196498); AtLAC17 (NP_200810), *Castanea mollissima* CmLAC (ACI46953); *Oryza sativa* OsLAC2 (Q8RYM9), OsLAC9 (Q6Z8L2); *Pinus taeda* PtLAC1 (AAK37823), PtLAC2 (AAK37824); *Ricinus communis* RcLAC (XP_002527130); *Solanum lycopersicum* ascorbate oxidase SlAOX (AAY47050); *Zea mays* ZmLAC3 (NP_001105915). This tree was constructed by the Neighbor-Joining method with *p*-distance. The number for each interior branch is the percent bootstraps value (1,000 replicates), and only values greater than 50% are shown. Scale bar indicates the estimated number of amino acid substitutions per site.
(TIF)

Figure S5 **QRT-PCR detection of transcription levels for** ***BnTT10*** **(A),** ***BrTT10*** **(B) and** ***BoTT10*** **(C) genes in various organs of black-seeded** ***B. napus*** **,** ***B. rapa*** **and** ***B. oleracea*** **, respectively.** Ro: root; Hy: hypocotyl; Co: cotyledon; St: stem; Le: leaf; Bu: bud; Fl: flower; SP: silique pericarp; 10, 15, 25, 30, 40, 45, 50, 52 and 55 D: seeds at 10, 15, 25, 30, 40, 45, 50, 52 and 55 days after flowering.
(TIF)

Figure S6 **Expression of** ***BnTT10*** **genes in the seeds of transgenic and control** ***B. napus*** **.** QRT-PCR analysis of overall *BnTT10* expression in seeds of T_1_ transgenic and control lines of Westar (A), Zhongyou821 (C) and Zhongshuang10 (E); qRT-PCR analysis of member-specific expression of *BnTT10* genes in seeds of transgenic and control lines of Westar (B), Zhongyou821 (D) and Zhongshuang10 (F). Error bars indicate SD.
(TIF)

Figure S7 ***In vitro*** **seed pigmentation of T_2_ transgenic and control of** ***B. napus*** **.** Siliques of T_2_ transgenic and control lines of *B. napus* were regularly sampled at 40, 50 and 55 DAF, and the pods opened 5 days later for observation under a low-power stereoscope. *In vitro* seed pigmentation of T_2_ transgenic and control *B. napus* cv. Zhongyou821 (A) and Zhongshuang10 (B) plants.
(TIF)

Figure S8 **Analysis of soluble (A) and insoluble (B) PAs measured after acid-catalyzed hydrolysis of seed coats from seeds of T_2_ transgenic and control** ***B. napus*** **cv. Zhongyou821 plants.** ZY-1, ZY-3 and ZY-17: transgenic lines with inhibited *BnTT10* expression; ZY-13: control lines with normal *BnTT10* expression. Values are means +/− SD from triplicate measurements in each sample.
(TIF)

Figure S9 **Analysis of soluble (A) and insoluble (B) PAs measured after acid-catalyzed hydrolysis of seed coats from seeds of T_2_ transgenic and control** ***B. napus*** **cv. Zhongshuang10 plants.** ZS-4, ZS-8 and ZS-12: transgenic lines with inhibited *BnTT10* expression; ZS-2: control lines with normal *BnTT10* expression. Values are means +/− SD from triplicate measurements in each sample.
(TIF)

Figure S10 **Lignin content in seed coats of transgenic and control** ***B. napus*** **cv. Zhongyou821 plants.** Lignin content was analyzed on seed coats from seeds of T_2_ transgenic and control lines using the acetyl bromide method. Values are means +/− SD from triplicate measurements in each sample.
(TIF)

Figure S11 **Lignin content in seed coats of transgenic and control** ***B. napus*** **cv. Zhongshuang10 plants.** Lignin content was analyzed on seed coats from seeds of T_2_ transgenic and control lines using the acetyl bromide method. Error bars indicate SD of three biological replicates.
(TIF)

Figure S12 **LC-UV-MS chromatograms of flavonoid compounds identified from seed coats extracts from control (A) and transgenic (B)** ***B. napus*** **cv. Zhongyou821 plants.**
(TIF)

Table S1 **Primers used in this study.**
(DOC)

Table S2 **Structure parameters of** ***BnTT10*** **,** ***BrTT10*** **and** ***BoTT10*** **genes.**
(DOC)

Table S3 **Percentages of full-length mRNA identities for** ***Brassica TT10*** **genes and** ***AtTT10*** **.**
(DOC)

Table S4 **Identities and positives between deduced** ***Brassica*** **TT10 proteins and AtTT10 (%).**
(DOC)
