# Supplementary material for: Gene Silencing of BnTT10 Family Genes Causes Retarded Pigmentation and Lignin Reduction in the Seed Coat of Brassica napus
Source: PLoS One. 2013 Apr 22;8(4):e61247. doi: 10.1371/journal.pone.0061247 (PMC3632561; doi:10.1371/journal.pone.0061247)
Supplement: Figure S1 — Alignment of the mRNA sequences of BnTT10 genes. Conservative similar residues were displayed in dark background. The 951-bp conserved fragment used for Southern hybridization and antisense suppression was highlighted by red line. (PDF) [file pone.0061247.s001.pdf]

```
           1        10        20        30        40        50        60        70        80        90
BnTT10-1   CCGCTTAACC-TTTGTTCCAATAAGCA-TTTGCTAAAGCACTCTCAAAATGTCACATCCCTTGTTCATTTACTTTCTAATCTCTCTTTCC
BnTT10-2   CCGCTTAACC-TTTGTTCCAATAAGCA-TTTGCTAAAGCACTCTCAAAATGTCACATCCCTTGTTCATTTACTTTCTAATCTCTCTTTCC
BnTT10-3     GATTAACCATCTGCCAGAATAATTATTTTACAAAAATTATTTCAAAATGCCACATTCTTTCTCTCATTTCTTCTTAATCTCTCTTTTC

                    100       110       120       130       140       150       160       170       180
BnTT10-1   CTCTACAGTAGTTGCACTGCACATCGCCACACATTCACGGTTAAGGAAGTTCCATATAAGAAACTGTGTAGCACGAAGAAGATTTTAACA
BnTT10-2   CTCTACAGTAGTTGCACTGCACATCGCTACACATTCACGGTTAAGGAAGTTCCATATAAGAAACTGTGTAGCACGAAGAAGATTTTAACA
BnTT10-3   CTTTACACCAAATGTATTGCGCATCGCTACACTTTCACGGTTAAGGAAGTTCCATATAACAAATTGTGTAGTACGAAGAAGATTCTGACC

                    190       200       210       220       230       240       250       260       270
BnTT10-1   GTTAATGGTCGGTTTCCTGGACAAACTTTAAAGGTTTACAAAGGAGACACCATTTACGTTAACGTTCGTAACCGAGCTAGTGAAAATATC
BnTT10-2   GTTAATGGTCAGTTTCCTGGACAAACTCTAAAGGTTTACAAAGGAGACACCATTTACGTTAACGTTCGTAACCGAGCTAGTGAAAATATC
BnTT10-3   GTTAACGGTCAGTTTCCTGGACCAGTTTTAAAGGTTTACAAAGGAGACACCATTTACGTCAACGTTCGTAACCGAGCTAGTGAAAACATC

                    280       290       300       310       320       330       340       350       360
BnTT10-1   ACCATGCATTGGCATGGTGTAGAGCAGCCGAGAAACCCATGGTCAGATGGACCAGAATACATCACACAATGCCCAATTCGACCTGGGTCT
BnTT10-2   ACCATGCATTGGCATGGTGTAGAGCAGCCGAGAAACCCATGGTCAGATGGACCAGAATACATCACACAATGCCCAATTCGACCTGGGTCT
BnTT10-3   ACAATGCATTGGCATGGTGTAGAGCAGCCGAGAAACCCGTGGTCAGATGGACCCGAATACATCACACAATGTCCGATTCGACCCGGAAGT

                    370       380       390       400       410       420       430       440       450
BnTT10-1   GATTTCATTTATGAAGTCATATTTTCCACCGAAGAGACGACTGTTTGGTGGCATGCTCATAGCTCTTGGACGCGTGCTACCGTACACGGT
BnTT10-2   GATTTCATGTATGAAGTCATATTTTCCACCGAAGACACGACTGTTTGGTGGCATGCTCATAGCTCTTGGACGCGTGCTACCGTACACGGT
BnTT10-3   GATTTTATGTATGAAGTCATCTTTTCCATCGAAGACGCAACTGTTTGGTGGCATGCGCATAGCTCTTGGACACGAGCCACTGTACATGGT

                    460       470       480       490       500       510       520       530       540
BnTT10-1   CTTATTTTTGTGTATCCTCGGCCCCCGAAATCCCTCCCTTTTCCAAAATCGGATCACGAAGTCCCTTTGATTTTTGGAGAGTGGTGGAAG
BnTT10-2   CTTATTTTTGTGTATCCTCGCCCCCCGAAATCCCTCCCTTTTCCAAAAGCTGATCATGAAGTCCCCTTGATTTTGGGAGAGTGGTGGAAG
BnTT10-3   CTTATATTCATATACCCTCGGCCCGGGACATTCCTCCCTTTCCCAAAGCCGGACCACGAAATTCCCTTAGTTCTTGGAGAGTGGTGGAAG

                    550       560       570       580       590       600       610       620       630
BnTT10-1   AAGGATGTGAGAGAAGTAGTGGAGCTGTTCATGAGGACAGGAGGTGACCCAAATGTTTCCGATGCTTTGACCATCAATGGACATCCTGGT
BnTT10-2   AAGGATGTGAGGGAAGTAGTGGAACTGTTCACGTGGACAGGAGGTGACCCAAATGTGTCCGATGCTTTGACCATCAATGGACATCCTGGT
BnTT10-3   GAAGATGTGAGAGAGGTAGTAGAGCAGTTCATAAGGACCGGAGCTGATCCTAATGTGTCCGATGCTTTGACTATCAATGGACATCCTGGT

                    640       650       660       670       680       690       700       710       720
BnTT10-1   TTCTTGTATCCTTGCTCTAAATCAGATACATTCGAGCTCATGGTGGAGAAAAGCAAAACCTATCGCATTCGGATGGTAAACGCCGCACTG
BnTT10-2   TTCTTGTATCCTTGCTCTAAATCAGATACATTCGAGTTCATGGTGGAGAAAAGCAAAACCTATCGCATTCGGATGGTAAACGCCGCAATG
BnTT10-3   TTCTTGTATCCTTGCTCTAATTCAGATACATTCGAGCTCACGGTAGAGAAGGGCAAAACCTATCGCATTCGGATGGTAAACGCCGCGATG

                    730       740       750       760       770       780       790       800       810
BnTT10-1   AACTTAATCCTCTTCTTCGCAATCGCAAAACACAACCTCACCGTGGTTGCCGCCGATGGCCACTACACCAAACCAATAAACGCTACTTAC
BnTT10-2   AACTTAATCCTCTTCTTCGCAATCGCAAAACACAATCTCACCGTGGTTGCCGCCGATGGCCACTACACCAAACCAATAAACGCTACTTAC
BnTT10-3   AACCTAATCTTCTTCTTCTCAATCGCAAAACACAGCCTCACCGTGGTTGCCGCCGATGGACACTACACCAAACCTCTAAACGCTACTTAC

                    820       830       840       850       860       870       880       890       900
BnTT10-1   ATCACCATCTCTCCAGGCCAAACGCTAGACTTGTTATTACACGCCGACCAAAACCCAAAAAGTACTTATTACATGGCTGCCAGAGCTTAC
BnTT10-2   ATCACCATCTCTCCAGGCCAAACGCTAGACTTGTTATTACACGCCGACCAAAATCCAAAAAGCACTTATTACATGGCCGCAAGAGCTTAC
BnTT10-3   ATCACCATATCTCCAGGTCAAACGCTGGACGTCTTATTACACGCGGACCAAAACCCAAAACGCACTTATTACATGGCAGCCAGAGCTTAC

                    910       920       930       940       950       960       970       980       990
BnTT10-1   CATAGCAACCCTAACATCAACTTCAACAACTCCACAACTATAGGGATCTTACGTTACACCTCTTC------------AACGTCATCGTCT
BnTT10-2   CATAGCAACCTTAACATCAGCTTCAACAACTCCACAACTATCGGGATCTTATGTTACAACTCTTCAAGTAAAACCAAAACGTCGTCGTCT
BnTT10-3   CACAGCAGCCCCGTCATCAGATTCAACAACTCCACAACTATCGGGATCTTACGTTACATCTCTTCAAGCAACGCCAAAACGTCATC----

                  1,000     1,010     1,020     1,030     1,040     1,050     1,060     1,070     1,080
BnTT10-1   TCAAAACGCTACCCAAACCTTCCTTATTACAATGACACATCAGCAGCGTTTAGATTCTTCACCAGTATCAAATGCTTATACTCCGGACAA
BnTT10-2   TCAAAACGTTACCCAAACCTTCCTTATTACAACGACACGTCAGCAGCTTTCGCATTCTTTACCAACATCACAAGCTTATACTCCGGACAA
BnTT10-3   --------TTATCCAAGCCTTCCTAATTATAATGACACGTCAGCAGCGTTCGGATTCTTTACGAACATCAAAAGCTTATACTCCGGACAA
```

```
          1,090     1,100     1,110     1,120     1,130     1,140     1,150     1,160     1,170
BnTT10-1  GTTCCCGTGAAGATCTCGCGTAGAATAATCTCGACGGTTTCAATAAACAACCTTATGTGTCCCAACAACTTGTGTGAAGGTCCAAACGGG
BnTT10-2  GTTCCCGTCAAGATCTCACGTAGAATAATCTCGACGGTTTCAATAAACCTTCTCATGTGTCCTAACAACTCGTGTGAAGGTCCAAACGGA
BnTT10-3  GTTCGAGTCAAAATCTCACGTAGAATAATCTCAACGGTTTCACTAAATCTTCTAGCGTGTCCTAACAACTCGTGCCAAGGTCCAAACGGG

          1,180     1,190     1,200     1,210     1,220     1,230     1,240     1,250     1,260
BnTT10-1  TCGAGACTAGCGGCGAGTATGAACAACATATCGTTCGTTACACCAAGTCACGTGGACATACTAAAAGCTTACTATTATCACATTAGGGGC
BnTT10-2  TCGAGACTAGCGGCGAGCATGAACAACATATCGTTCGTTACACCGAGTCACGTGGACATACTAAAAGCTTACTATTATCACATTAAGGGA
BnTT10-3  TTGAGATTAGCGGCTAGTATGAACAACATATCATTCGTCACACCAAACCACGTAGACATACTAAAAGCTTATTACCTTCACATTAAGGGC

          1,270     1,280     1,290     1,300     1,310     1,320     1,330     1,340     1,350
BnTT10-1  GTTTACGGAACGCGGTTTCCGGAGTTTCCACCGCTGGTTTTCAACTTTACCGCGGATGATCAACCGTTGTTTTTGCAGACTCCAAGATTT
BnTT10-2  GTTTACGGAACGCGGTTTCCAGAGTTTCCACCGCTCGTTTTCAACTTTACCGCGGATGATCAACCGTTGTTTTTGCAGACTCCAAGACTT
BnTT10-3  GTTTACGGAACGCGGTTTCCTGCGTTTCCGCCTCTGGTTTTCAATTTTACCGCGGATGATCAACCGTTGATTTTGCAGACTTCGAGACTT

          1,360     1,370     1,380     1,390     1,400     1,410     1,420     1,430     1,440
BnTT10-1  GCTACGGAGGTGAAAATACTTAAGTTTGGGGAAAGTGTTGAGATTGTTCTCCAAGGGACGAGTTTAGTTGGTGGTGGAATCGATCATCCC
BnTT10-2  GCTACGGAGGTGAAAATACTTAAGTTTGGGGAAAGTGTTGAGATTGTTCTCCAAGGGACGAGTTTAGTTGGTGGTGGAATCGATCATCCC
BnTT10-3  GCTACGGAAGTAAAGATGATTAAGTACGGAGAAGCGGTTGAAACGGTTTTTCAAGGGACGAGTTTAGGTGGTGGTGGAATCGACCACCCC

          1,450     1,460     1,470     1,480     1,490     1,500     1,510     1,520     1,530
BnTT10-1  ATGCATCTCCATGGTTTTAGTTTCTACGAGGTTGGTGTCGGGTTTGGTAACTATAACGTAACTGAAGATC---CGTCGAACTATAATCTC
BnTT10-2  ATGCATCTCCATGGTTTTAGTTTCTACATGGTTGGTGTCGGGTTTGGTAACTATAACGTAACTGAAGCTC---CGTCGAACTATAACCTC
BnTT10-3  ATGCATTTGCATGGTTTTAGCTTCTATATTGTTGGTTTTGGGTTTGGTAACTATAACGTAACGAAAGATCCGTCGTCGAACTATAATCTC

          1,540     1,550     1,560     1,570     1,580     1,590     1,600     1,610     1,620
BnTT10-1  AAAGATCCTCCGTACATAAACACTGCGACTGTGCCAAGAAACGGTTGGGTCGCTATCAGATTCATAGCTGATAATCCTGGGGTATGGTTC
BnTT10-2  AAAGATCCTCCGTACAAAAATACTGCGACTGTGCCAAGAAACGGTTGGGTCGCTATCAGATTCATAGCTGACAATCCTGGGGTATGGTTC
BnTT10-3  AAAGATCCTCCGTATAGAAATACGGCGACTGTGCCAAGAAACGGTTGGATCGCTATCAGATTCATAGCTAACAATCCTGGGGTTTGGTTC

          1,630     1,640     1,650     1,660     1,670     1,680     1,690     1,700     1,710
BnTT10-1  ATGCACTGCCACTTTGATAGACATCTCACGTGGGGAATGAAAGTTGTCTTCATTGTCATGAATGGAAGAGGACTAAACCAGCAGATTCTG
BnTT10-2  ATGCACTGCCACTTTGATAGACATCTCACGTGGGGAATGAAAGTGGTCTTCATTGTCAAGAATGGAAGAGGACTAAACCAGCAGATTCTG
BnTT10-3  ATGCATTGTCACTTTGATAGACATCTCACGTGGGGTATGAACGTTGTCTTCATTGTCGAGAACGGCAGAGGAATAAACCAGCAGATGCTG

          1,720     1,730     1,740     1,750     1,760     1,770     1,780     1,790     1,800
BnTT10-1  CCTCCACCTCCTAACTTGCCTCCTTGTTACTAATCAATAATCCAATTATGTAAAAATCACTATCATGTCTCTTAACCAAAAATGACATTA
BnTT10-2  CCTCCACCTCCTGACTTGCCTCCTTGTTACTAATTAATAATCCAATTATGT-AAAATCACTATCATGTCT-----------------TTA
BnTT10-3  CCTCCACCGCCTGACTTGCCGCCTTGTTACT----AATAATTCAACTGT-----------TACCAATT-------------------TGA

          1,810     1,820     1,830     1,840     1,850     1,860     1,870     1,880     1,890
BnTT10-1  ATCTATTGTAAATTATTGT------TGTAATAACCTAAATTTACTCCAATGTAATAATCCTTAAACTTTGTGTGAATTAATTTCCACAAG
BnTT10-2  TTCTGTTGTAAATTATTGT------TGTAATAACCTAAATTTACTCCAATGTAATAATCCTAAAACTTTGTGTGAATTAATTTCCACAAG
BnTT10-3  GTATAATGAAAGTCATTGTAACTGATGTAATAAACTAAATTTA--CCAAAGATATAATCCTTAGTAATCAT---AAGGAAGACTCCCAAG

          1,900     1,910     1,920          1,935
BnTT10-1  TTGATTAAAGAATAAGAAGAAGCATTACTAAAAAAA
BnTT10-2  TTGATT-AAGAATAAGAAGAAGCATTACTAAAAA
BnTT10-3  T---CCCAACAAAAAAAGGTGATAATACTAAACCAAACCGTTGTC
```
