# Supplementary figures and images for: Gene Silencing of BnTT10 Family Genes Causes Retarded Pigmentation and Lignin Reduction in the Seed Coat of Brassica napus
Source: PLoS One. 2013 Apr 22;8(4):e61247. doi: 10.1371/journal.pone.0061247 (PMC3632561; doi:10.1371/journal.pone.0061247)

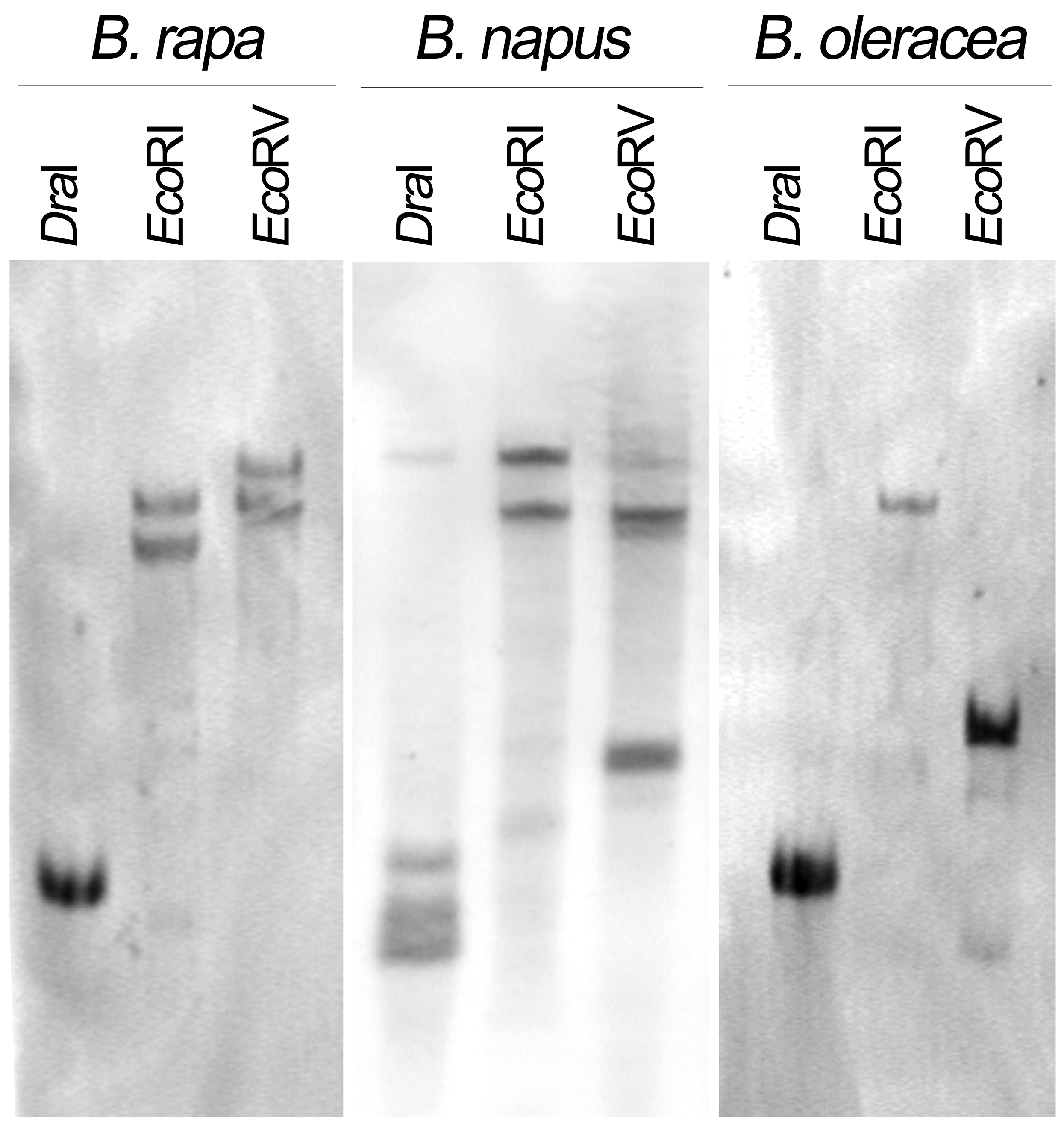

Supplement: Figure S2 — Southern blot hybridization detection of TT10 genes in B. napus , B. oleracea and B. rapa . (TIF) [file pone.0061247.s002.tif]

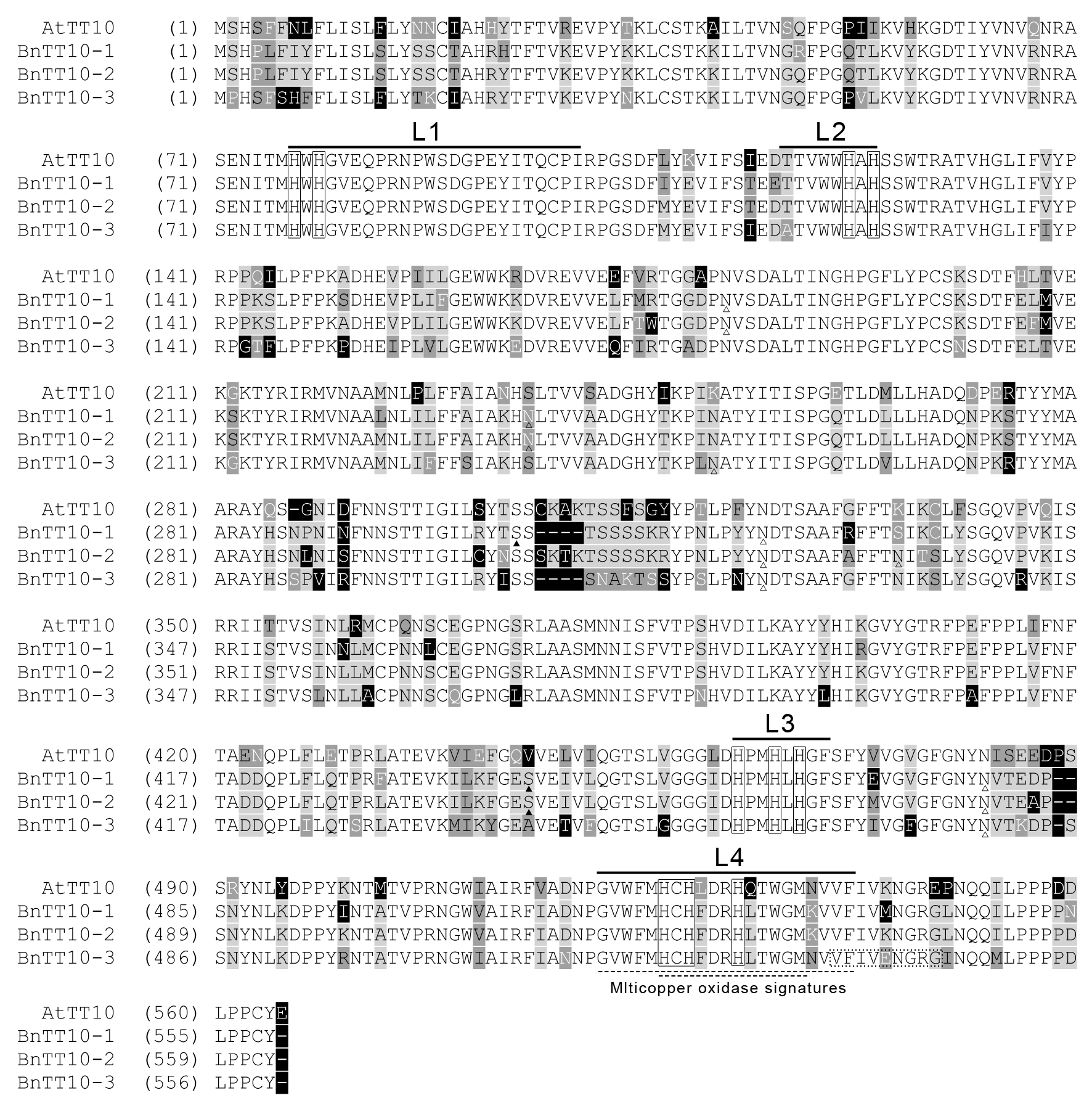

Supplement: Figure S3 — Alignment of the amino acid sequences of BnTT10 proteins. BrTT10 and BoTT10-1 proteins share high identity with BnTT10 proteins and were not shown in the alignment. Non-similar, weakly similar, block of similar, conservative/strongly similar residues are displayed in dark, dark gray, gray and light gray background, respectively. The four His-rich copper binding domains, L1-L4, are highlighted by line segments, and the amino acids involved in binding copper are boxed. Two predicted multicopper oxidase signature 1 and 2 are identified by single and double dotted lines, respectively. (TIF) [file pone.0061247.s003.tif]

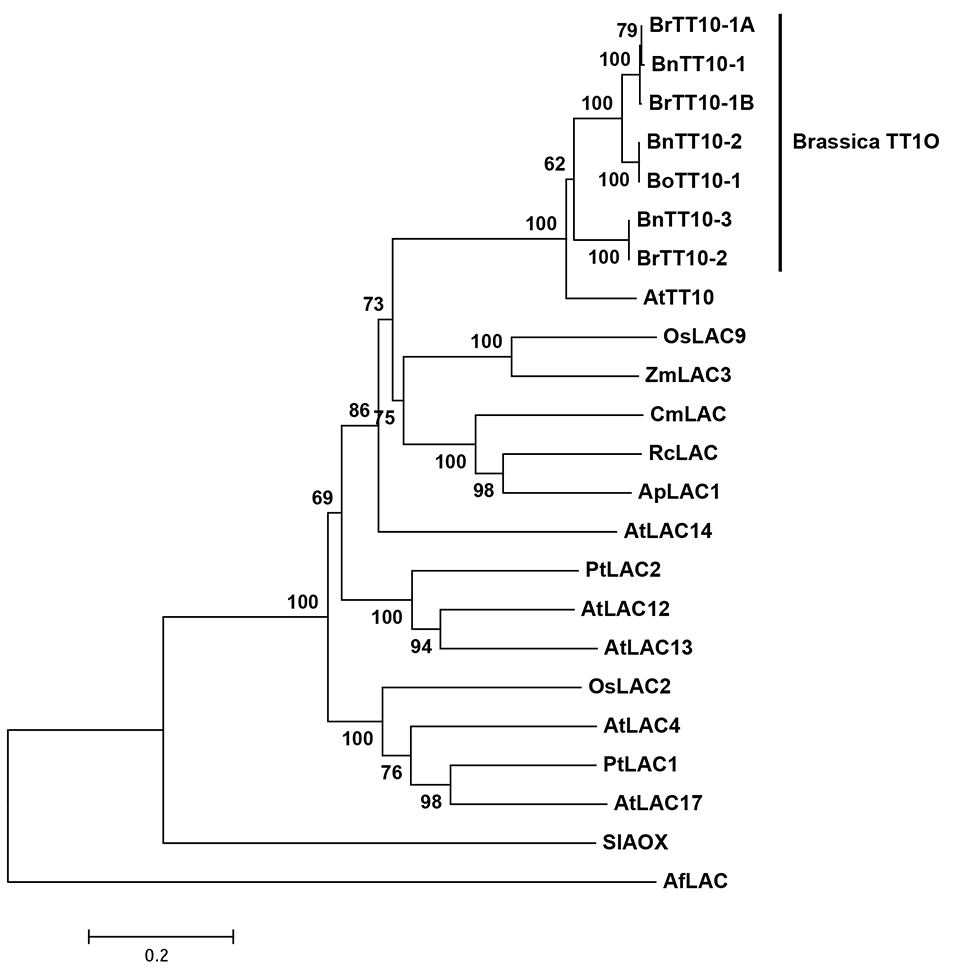

Supplement: Figure S4 — Phylogenetic relationship of inferred Brassica TT10 proteins and other plant laccases. Acer pseudoplatanus ApLAC1 (AAB09228); Aspergillus flavus AfLAC (XP_002378028); Arabidopsis thaliana AtTT10 (NP_199621), AtLAC4 (NP_565881), AtLAC12 (NP_196158), AtLAC13 (NP_196330), AtLAC14 (NP_196498); AtLAC17 (NP_200810), Castanea mollissima CmLAC (ACI46953); Oryza sativa OsLAC2 (Q8RYM9), OsLAC9 (Q6Z8L2); Pinus taeda PtLAC1 (AAK37823), PtLAC2 (AAK37824); Ricinus communis RcLAC (XP_002527130); Solanum lycopersicum ascorbate oxidase SlAOX (AAY47050); Zea mays ZmLAC3 (NP_001105915). This tree was constructed by the Neighbor-Joining method with p-distance. The number for each interior branch is the percent bootstraps value (1,000 replicates), and only values greater than 50% are shown. Scale bar indicates the estimated number of amino acid substitutions per site. (TIF) [file pone.0061247.s004.tif]

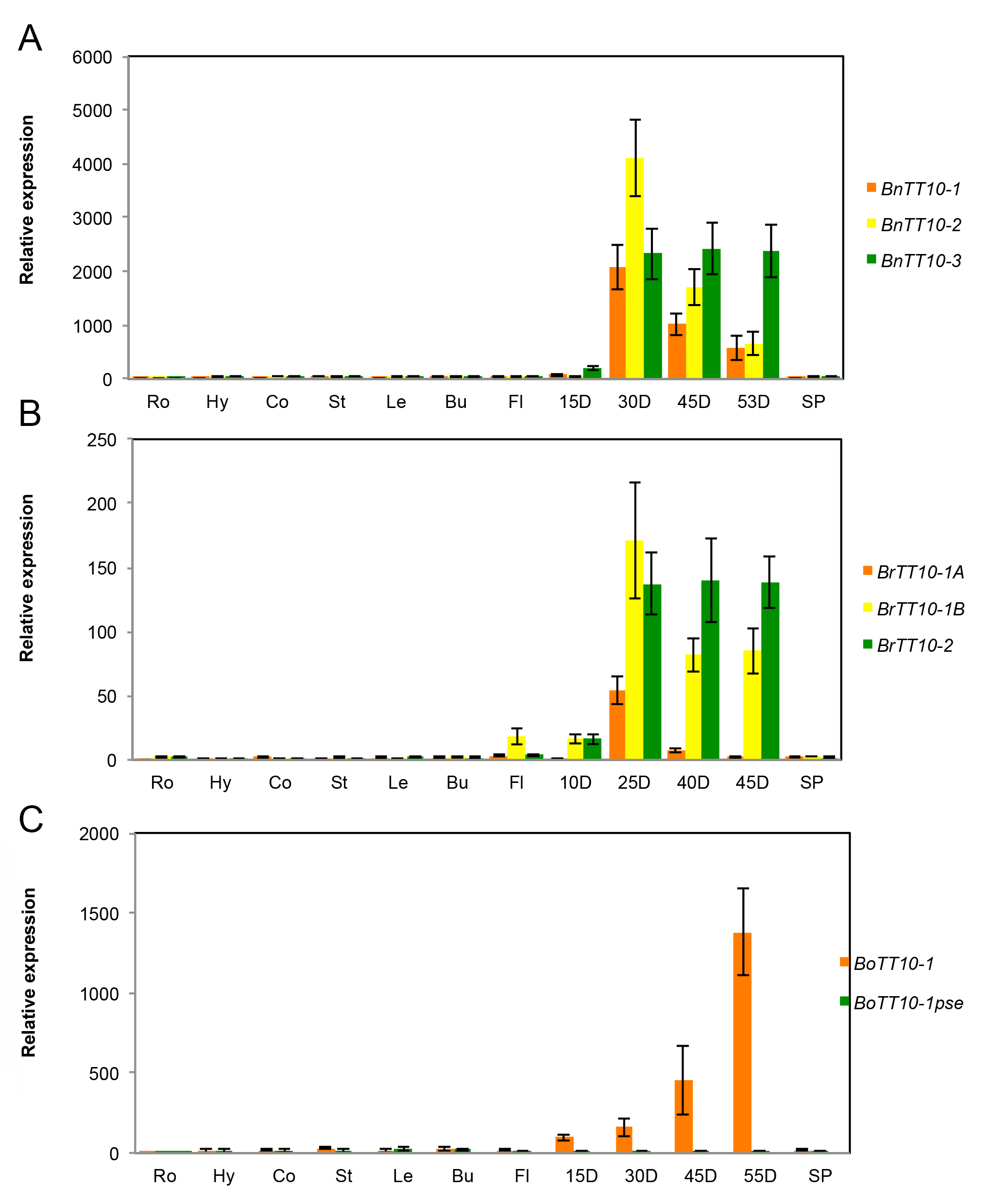

Supplement: Figure S5 — QRT-PCR detection of transcription levels for BnTT10 (A), BrTT10 (B) and BoTT10 (C) genes in various organs of black-seeded B. napus , B. rapa and B. oleracea , respectively. Ro: root; Hy: hypocotyl; Co: cotyledon; St: stem; Le: leaf; Bu: bud; Fl: flower; SP: silique pericarp; 10, 15, 25, 30, 40, 45, 50, 52 and 55 D: seeds at 10, 15, 25, 30, 40, 45, 50, 52 and 55 days after flowering. (TIF) [file pone.0061247.s005.tif]

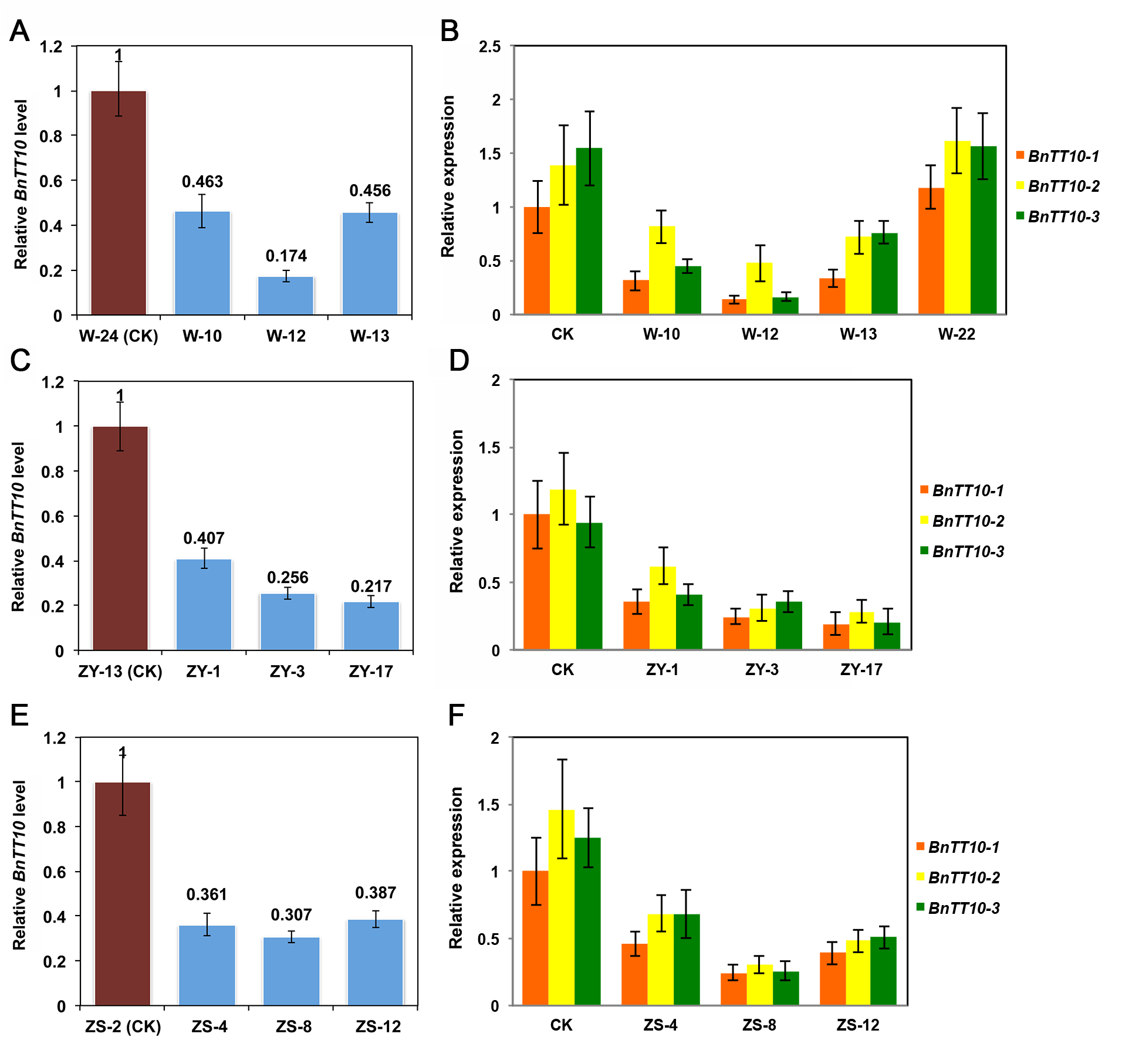

Supplement: Figure S6 — Expression of BnTT10 genes in the seeds of transgenic and control B. napus . QRT-PCR analysis of overall BnTT10 expression in seeds of T1 transgenic and control lines of Westar (A), Zhongyou821 (C) and Zhongshuang10 (E); qRT-PCR analysis of member-specific expression of BnTT10 genes in seeds of transgenic and control lines of Westar (B), Zhongyou821 (D) and Zhongshuang10 (F). Error bars indicate SD. (TIF) [file pone.0061247.s006.tif]

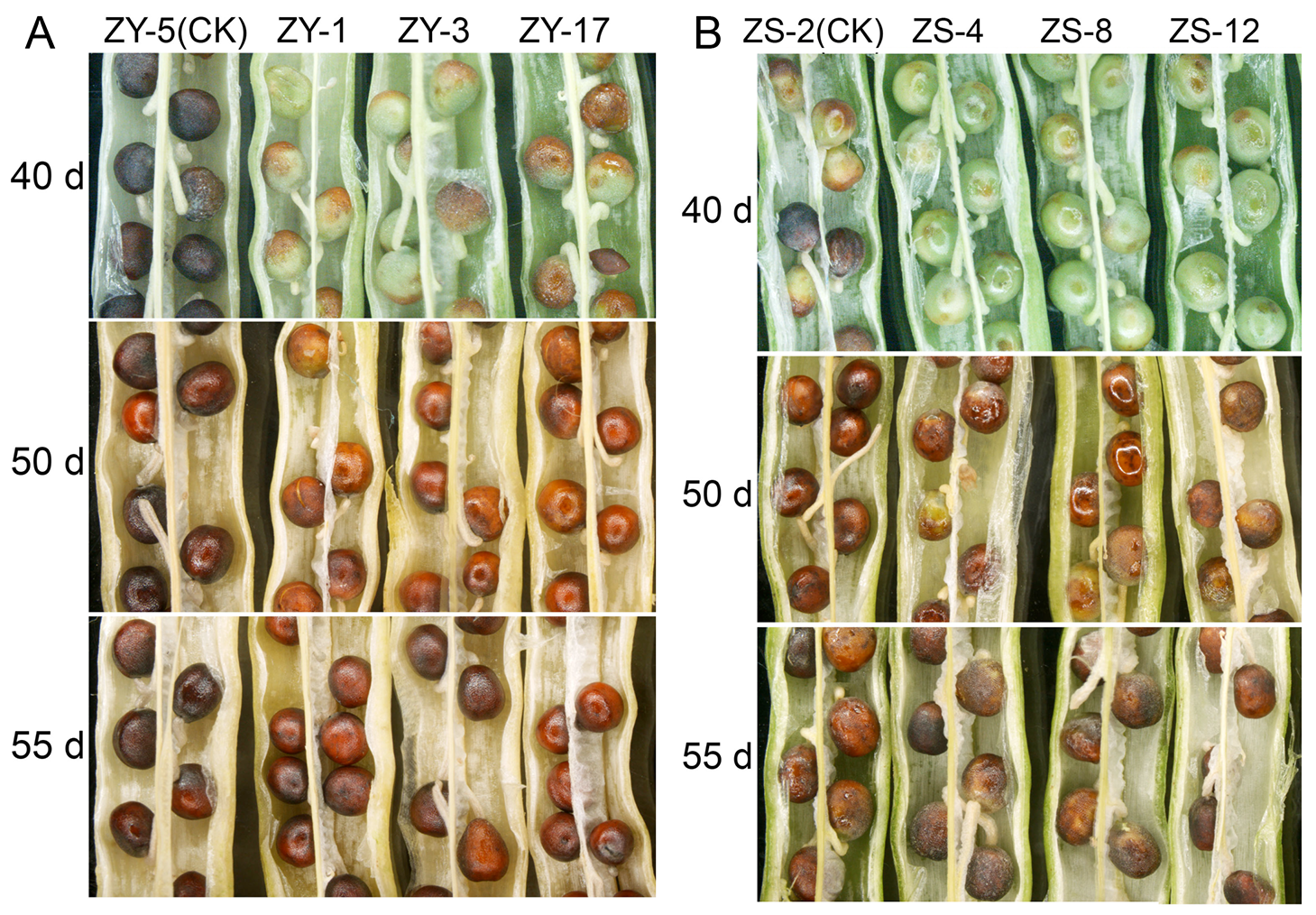

Supplement: Figure S7 — In vitro seed pigmentation of T2 transgenic and control of B. napus . Siliques of T2 transgenic and control lines of B. napus were regularly sampled at 40, 50 and 55 DAF, and the pods opened 5 days later for observation under a low-power stereoscope. In vitro seed pigmentation of T2 transgenic and control B. napus cv. Zhongyou821 (A) and Zhongshuang10 (B) plants. (TIF) [file pone.0061247.s007.tif]

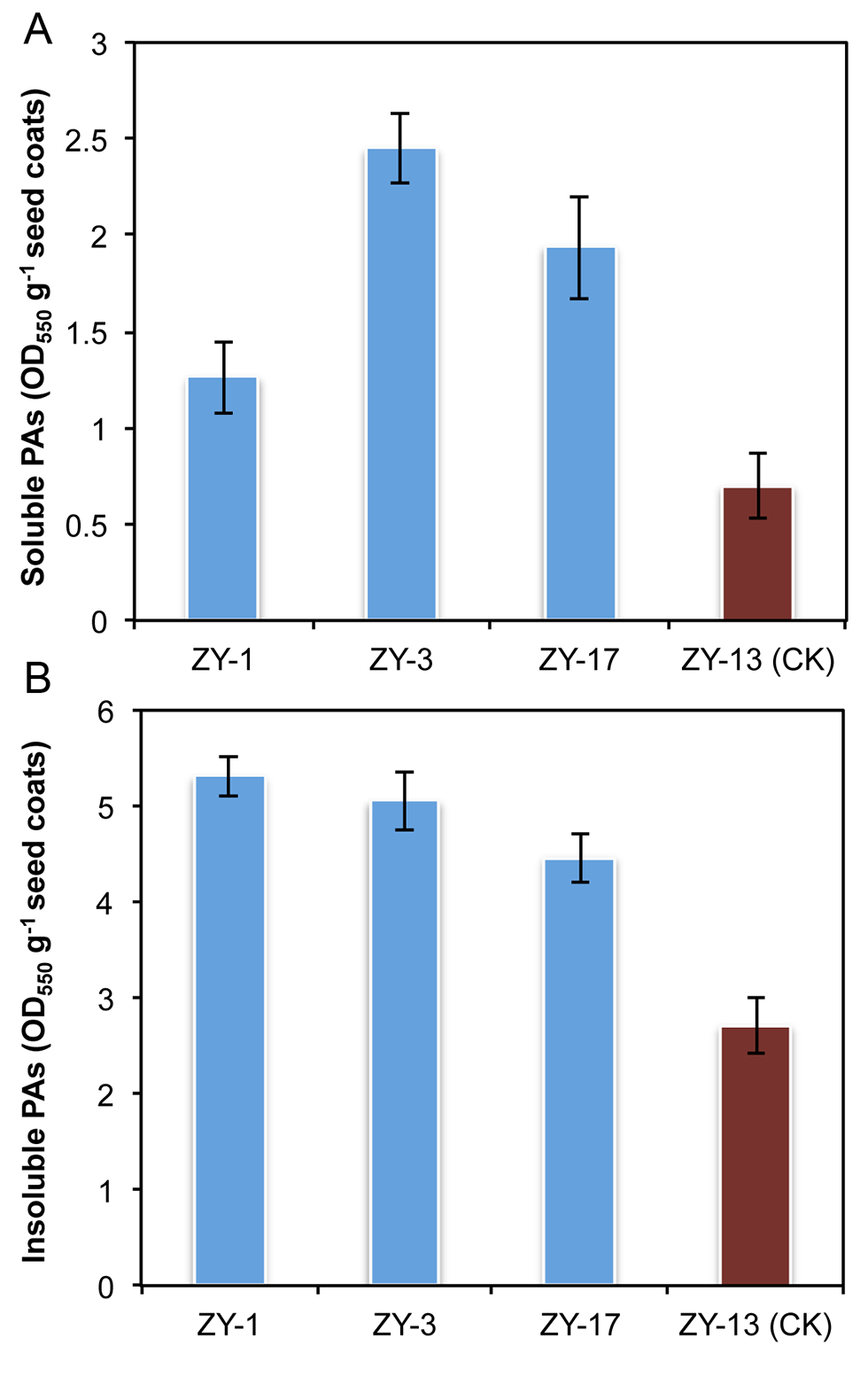

Supplement: Figure S8 — Analysis of soluble (A) and insoluble (B) PAs measured after acid-catalyzed hydrolysis of seed coats from seeds of T2 transgenic and control B. napus cv. Zhongyou821 plants. ZY-1, ZY-3 and ZY-17: transgenic lines with inhibited BnTT10 expression; ZY-13: control lines with normal BnTT10 expression. Values are means +/− SD from triplicate measurements in each sample. (TIF) [file pone.0061247.s008.tif]

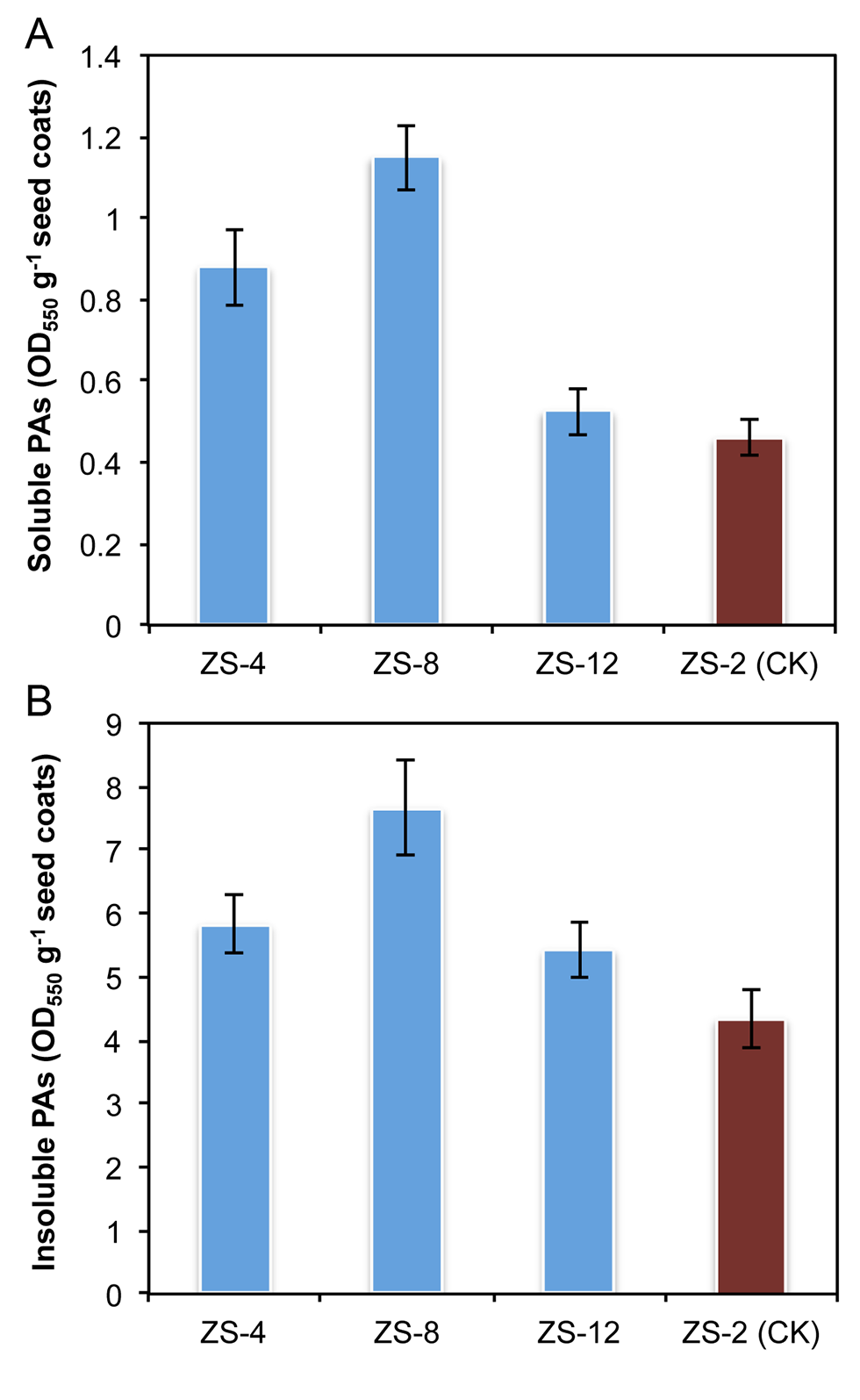

Supplement: Figure S9 — Analysis of soluble (A) and insoluble (B) PAs measured after acid-catalyzed hydrolysis of seed coats from seeds of T2 transgenic and control B. napus cv. Zhongshuang10 plants. ZS-4, ZS-8 and ZS-12: transgenic lines with inhibited BnTT10 expression; ZS-2: control lines with normal BnTT10 expression. Values are means +/− SD from triplicate measurements in each sample. (TIF) [file pone.0061247.s009.tif]

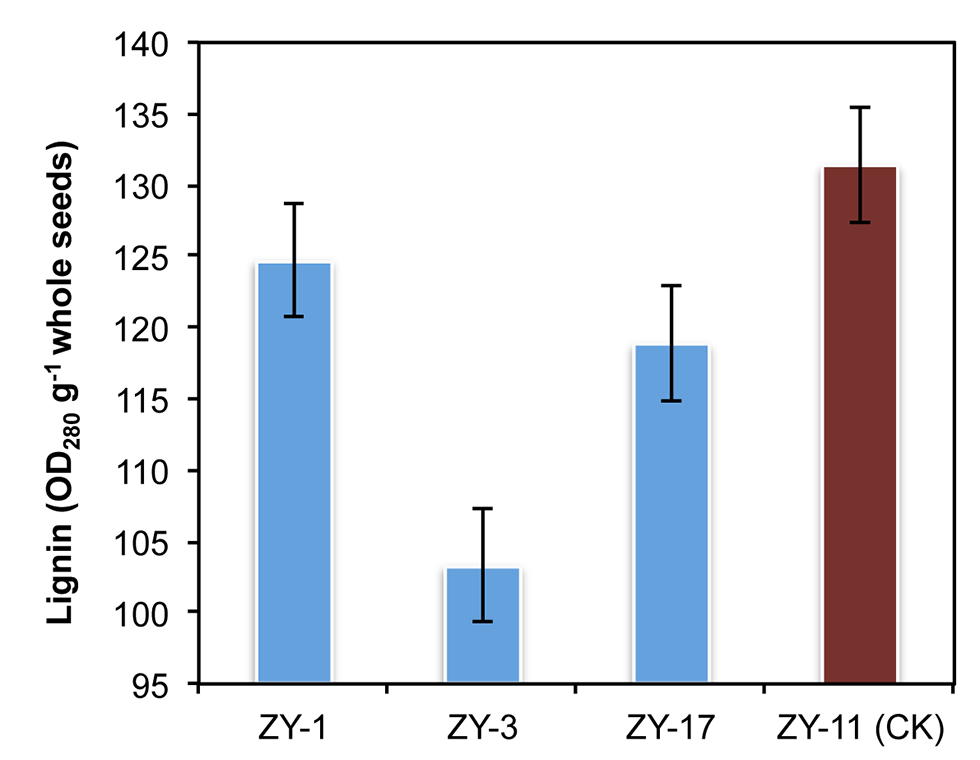

Supplement: Figure S10 — Lignin content in seed coats of transgenic and control B. napus cv. Zhongyou821 plants. Lignin content was analyzed on seed coats from seeds of T2 transgenic and control lines using the acetyl bromide method. Values are means +/− SD from triplicate measurements in each sample. (TIF) [file pone.0061247.s010.tif]

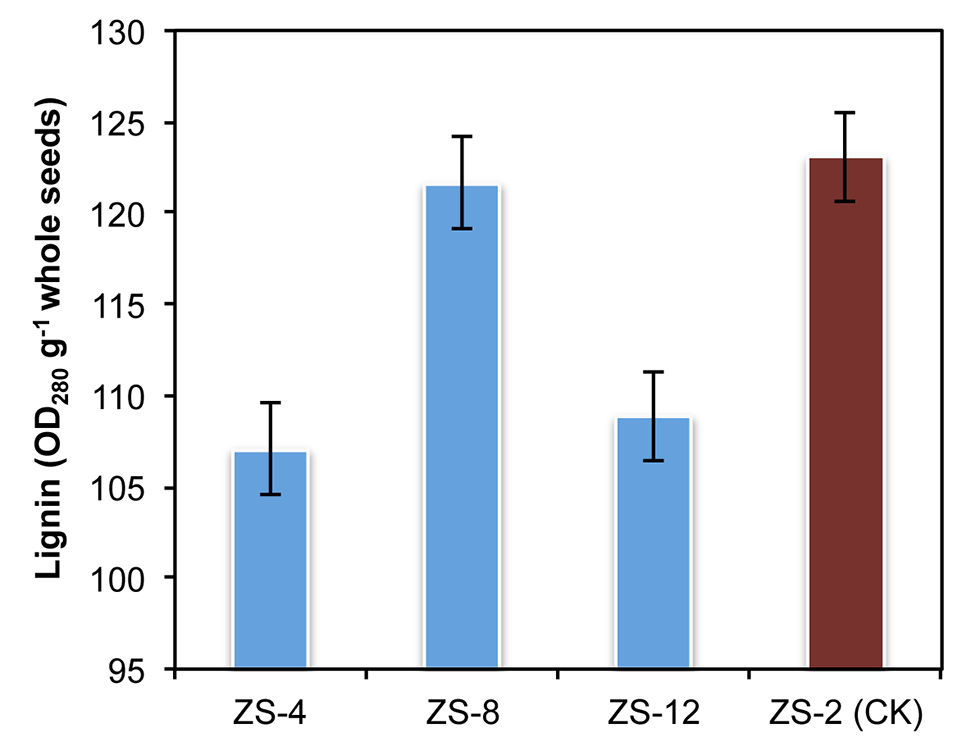

Supplement: Figure S11 — Lignin content in seed coats of transgenic and control B. napus cv. Zhongshuang10 plants. Lignin content was analyzed on seed coats from seeds of T2 transgenic and control lines using the acetyl bromide method. Error bars indicate SD of three biological replicates. (TIF) [file pone.0061247.s011.tif]

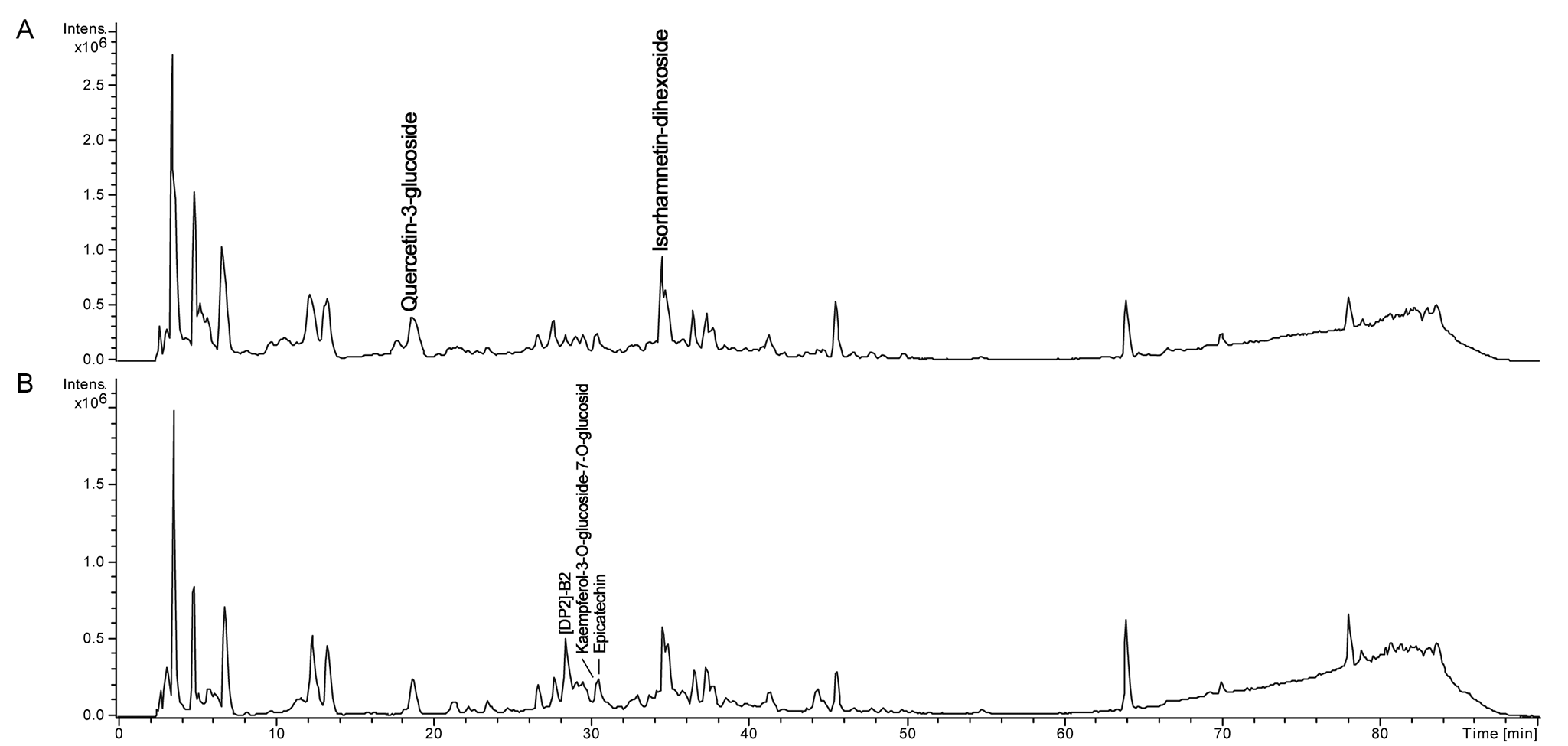

Supplement: Figure S12 — LC-UV-MS chromatograms of flavonoid compounds identified from seed coats extracts from control (A) and transgenic (B) B. napus cv. Zhongyou821 plants. (TIF) [file pone.0061247.s012.tif]
