# Supplementary material for: Gene Silencing of BnTT10 Family Genes Causes Retarded Pigmentation and Lignin Reduction in the Seed Coat of Brassica napus
Source: PLoS One. 2013 Apr 22;8(4):e61247. doi: 10.1371/journal.pone.0061247 (PMC3632561; doi:10.1371/journal.pone.0061247)
Supplement: Table S1 — Primers used in this study. (DOC) [file pone.0061247.s013.doc]

**Table S1** Primers used in this study.

| Primer | Sequence of Primers |
| --- | --- |
| GeneRacer 5'P | 5'-CGACTGGAGCACGAGGACACTGA-3' |
| GeneRacer 5'NP | 5'-GGACACTGACATGGACTGAAGGAGTA-3' |
| GeneRacer 3'P | 5'-GCTGTCAACGATACGCTACGTAACG-3' |
| GeneRacer 3'NP | 5'-CGCTACGTAACGGCATGACAGTG-3' |
| FTT10-31 | 5'-CCTATGCATCTCCATGGTTTTAGCTT-3' |
| FTT10-32 | 5'-TTCTATGTGGTTGGAGTAGGGTTCGG-3' |
| RTT10-51 | 5'-CTATGCGCGTGCCACCAAACAGT-3' |
| RTT10-52 | 5'-TGCCACCAAACAGTCGTGTCTTC-3' |
| FBNTT10 | 5'-GGATCCAACCTTTGTTCCAATAAGCATTTGC-3' |
| FBRTT10 | 5'-GGATCCAACCATCTGCCAGAATAATTATTTTAC-3' |
| FB0TT10 | 5'-GGATCCCCGCTTAACCTTTGTTCCAATAAGC-3' |
| RBNTT10 | 5'-GAGCTCAGTAATGCTTCTTCTTATTCTTAATCAAC-3' |
| RBRTT10 | 5'-GAGCTCAGTAATGCTTCTTCTTATTCTTTAATCAAC-3' |
| RBRTT10-I | 5'-GAGCTCGGTAAATTTAGTTTATTACATCAGTTAC-3' |
| RBOTT10 | 5'-GAGCTCAATTCACACAAAGTTTTACGATTATTACA-3' |
| FTT10A | 5'-GAGCTCATACATTCGAGTTCATGGTGGAG-3' |
| RTT10A | 5'-GGATCCCAGGATTGTCAGCTATGAATCTG-3' |
| F18qS | 5'-AACCAAACATCTCACGACAC-3' |
| R18qS | 5'-GCAAGACCGAAACTCAAAG-3' |
| FBnT10Q | 5'-GACATCCTGGTTTCTTGTATC-3' |
| RBnT10Q | 5'-GTTTACCATCCGAATGCGATA-3' |
| FBnT10-1Q | 5'-AGATTCTTCACCAGTATCAAATGCTTAT-3' |
| RBnT10-1Q | 5'-CGTTTGGACCTTCACACAAGT-3' |
| FBnT10-2Q | 5'-GATACATTCGAGTTCATGGTGGAG-3' |
| RBnT10-2Q | 5'-CGTGTCGTTGTAATAAGGAAGGTT-3' |
| FBnT10-3Q | 5'-GGAGAGTGGTGGAAGGAAGA-3' |
| RBnT10-3Q | 5'-GCGATTGAGAAGAAGAAGATTAGGT-3' |
| FBrT10-1BQ | 5'-AACTTGCCTCCTTGTTACTGATC-3' |
| RBrT10-1BQ | 5'-TCTTCTTATTCTTTAATCAACCTGTGGA-3' |
| FBoT10-1pseQ | 5'- CCATGTCAGATGGACCAGAATAC -3' |
| RBoT10-1pseQ | 5'-TTTAGGGCAAGGATACAAGAAACC-3' |
