# Supplementary material for: Gene Silencing of BnTT10 Family Genes Causes Retarded Pigmentation and Lignin Reduction in the Seed Coat of Brassica napus
Source: PLoS One. 2013 Apr 22;8(4):e61247. doi: 10.1371/journal.pone.0061247 (PMC3632561; doi:10.1371/journal.pone.0061247)
Supplement: Table S2 — Structure parameters of BnTT10 , BrTT10 and BoTT10 genes. (DOC) [file pone.0061247.s014.doc]

**Table S2** Structure parameters of *BnTT10*, *BrTT10* and *BoTT10* genes.

| Group | Gene name | DNA  (bp) | cDNA  (bp) | 5'UTR  (bp) | Coding region  (bp) | Position of introns (bp) | | | | | 3'UTR  (bp) |
| --- | --- | --- | --- | --- | --- | --- | --- | --- | --- | --- | --- |
|  |  |  |  |  |  | 1 | 2 | 3 | 4 | 5 |  |
| I | *BnTT10-3* | 2503 | 1879 | 46 | 1683 | 128-219 | 372-451 | 697-817 | 947-1153 | 2096-2219 | 150 |
|  | *BrTT10-2* | 2503 | 1879 | 46 | 1683 | 128-219 | 372-451 | 697-817 | 947-1153 | 2096-2219 | 150 |
| II | *BnTT10-1* | 4682 | 1896 | 46 | 1680 | 128-213 | 366-617 | 863-935 | 1065-3085 | 4025-4378 | 170 |
|  | *BnTT10-2* |  | 1889 | 46 | 1692 |  |  |  |  |  | 151 |
|  | *BrTT10-1A* | 3662 | 1896 | 46 | 1680 | 128-213 | 366-617 | 863-935 | 1065-2058 | 2998-3358 | 170 |
|  | *BrTT10-1B* | 3712 | 1896 | 46 | 1680 | 128-213 | 366-617 | 863-935 | 1065-2108 | 3048-3408 | 170 |
|  | *BoTT10-1* | 5475 | 1889 | 46 | 1692 | 128-213 | 366-600 | 846-917 | 1047-3853 | 4805-5190 | 151 |
|  | *BoTT10-1pse* |  | 1887 | 46 | 471 |  |  |  |  |  | 1370 |
