# Supplementary material for: Gene Silencing of BnTT10 Family Genes Causes Retarded Pigmentation and Lignin Reduction in the Seed Coat of Brassica napus
Source: PLoS One. 2013 Apr 22;8(4):e61247. doi: 10.1371/journal.pone.0061247 (PMC3632561; doi:10.1371/journal.pone.0061247)
Supplement: Table S3 — Percentages of full-length mRNA identities for Brassica TT10 genes and AtTT10 . (DOC) [file pone.0061247.s015.doc]

**Table S3** Percentages of full-length mRNA identities for *Brassica TT10* genes (excluding *BoTT10-1pse*) and *AtTT10* (%).

| Genes | *BnTT10-3* | *BrTT10-2* | *BnTT10-1* | *BrTT10-1A* | *BrTT10-1B* | *BnTT10-2* | *BoTT10-1* |
| --- | --- | --- | --- | --- | --- | --- | --- |
| *AtTT10* | 81.2/82.7 | 79.8/82.5 | 79.4/82.9 | 79.2/83.1 | 79.1/83.1 | 80.2/83.6 | 80.2/83.6 |
| *BnTT10-3* | -- | 99.7/99.8 | 83.5/85.5 | 83.5/85.8 | 83.6/85.9 | 84.6/86.3 | 84.6/86.3 |
| *BrTT10-2* |  | -- | 83.3/85.2 | 83.3/85.6 | 83.4/85.6 | 84.3/85.3 | 84.3/85.5 |
| *BnTT10-1* |  |  | -- | 99.8/99.6 | 99.3/99.2 | 94.6/95.0 | 94.6/95.0 |
| *BrTT10-1A* |  |  |  | -- | 99.5/99.5 | 94.7/95.3 | 94.7/95.3 |
| *BrTT10-1B* |  |  |  |  | -- | 94.6/95.4 | 94.6/95.4 |
| *BnTT10-2* |  |  |  |  |  | -- | 100.0/100.0 |
