# Supplementary material for: Gene Silencing of BnTT10 Family Genes Causes Retarded Pigmentation and Lignin Reduction in the Seed Coat of Brassica napus
Source: PLoS One. 2013 Apr 22;8(4):e61247. doi: 10.1371/journal.pone.0061247 (PMC3632561; doi:10.1371/journal.pone.0061247)
Supplement: Table S4 — Identities and positives between deduced Brassica TT10 proteins and AtTT10 (%). (DOC) [file pone.0061247.s016.doc]

**Table S4** Identities and positives between deduced *Brassica* TT10 proteins (excluding BoTT10-2) and AtTT10 (%).

| Proteins | BnTT10-3 | BrTT10-2 | BnTT10-1 | BrTT10-1A | BrTT10-1B | BnTT10-2 | BoTT10-1 |
| --- | --- | --- | --- | --- | --- | --- | --- |
|  | Ide. Pos | Ide. Pos | Ide. Pos | Ide. Pos | Ide. Pos | Ide. Pos | Ide. Pos |
| AtTT10 | 80.4 86.0 | 80.4 86.0 | 81.1 86.4 | 80.9 86.2 | 81.1 86.5 | 83.0 87.4 | 83.0 87.4 |
| BnTT10-3 | -- | 100.0 100.0 | 83.9 88.6 | 83.8 88.4 | 83.9 88.8 | 85.5 89.0 | 85.5 89.0 |
| BrTT10-2 |  | -- | 83.9 88.6 | 83.8 88.4 | 83.9 88.8 | 85.5 89.0 | 85.5 89.0 |
| BnTT10-1 |  |  | -- | 99.8 99.8 | 99.5 99.6 | 94.5 95.6 | 94.5 95.6 |
| BrTT10-1A |  |  |  | -- | 99.3 99.5 | 94.1 95.2 | 94.1 95.2 |
| BrTT10-1B |  |  |  |  | -- | 94.3 95.6 | 94.3 95.6 |
| BnTT10-2 |  |  |  |  |  | -- | 100.0 100.0 |

Ide.: identities；Pos.: positives
